# KDM2 proteins constrain transcription from CpG island gene promoters independently of their histone demethylase activity

**DOI:** 10.1093/nar/gkz607

**Published:** 2019-07-31

**Authors:** Anne H Turberfield, Takashi Kondo, Manabu Nakayama, Yoko Koseki, Hamish W King, Haruhiko Koseki, Robert J Klose

**Affiliations:** 1 Department of Biochemistry, University of Oxford, Oxford, OX1 3QU, UK; 2 Laboratory for Developmental Genetics, RIKEN Center for Integrative Medical Sciences, Yokohama, Japan; 3 Department of Technology Development, Kazusa DNA Research Institute, Kisarazu, Japan; 4 CREST, Japan Science and Technology Agency, Kawaguchi, Japan

## Abstract

CpG islands (CGIs) are associated with the majority of mammalian gene promoters and function to recruit chromatin modifying enzymes. It has therefore been proposed that CGIs regulate gene expression through chromatin-based mechanisms, however in most cases this has not been directly tested. Here, we reveal that the histone H3 lysine 36 (H3K36) demethylase activity of the CGI-binding KDM2 proteins contributes only modestly to the H3K36me2-depleted state at CGI-associated gene promoters and is dispensable for normal gene expression. Instead, we discover that KDM2 proteins play a widespread and demethylase-independent role in constraining gene expression from CGI-associated gene promoters. We further show that KDM2 proteins shape RNA Polymerase II occupancy but not chromatin accessibility at CGI-associated promoters. Together this reveals a demethylase-independent role for KDM2 proteins in transcriptional repression and uncovers a new function for CGIs in constraining gene expression.

## INTRODUCTION

The functionality of complex multicellular organisms is underpinned by the creation of diverse cell types from a common genetic DNA blueprint. This is achieved during development by cells acquiring and maintaining cell type-specific gene expression programmes. At the most basic level, this relies on the control of RNA polymerase II (RNAPII)-mediated transcription by transcription factors (1). However, it has also become clear that chromatin structure and its chemical modification can profoundly affect how transcription initiates from promoters and how gene expression is controlled (2,3).

One such chemical modification of chromatin occurs on DNA where a methyl group is added to the five position of each cytosine in the context of CpG dinucleotides. CpG methylation is pervasive in mammalian genomes and is generally associated with transcriptional repression, particularly of repetitive and parasitic DNA elements (4,5). However, short CpG-rich regions of the genome, called CpG islands (CGIs), remain free of DNA methylation and are associated with the majority of mammalian gene promoters (6,7). CGIs have been proposed to regulate gene expression (8,9) through a family of ZF-CxxC DNA binding domain-containing proteins that recognize non-methylated DNA and occupy CGIs (10–13). Interestingly, most ZF-CxxC domain-containing proteins possess histone modifying activities or are part of large chromatin modifying complexes, suggesting that these factors regulate gene expression through chromatin (14). However, in most cases the contribution of chromatin-based mechanisms to CGI-dependent gene regulation remains untested.

The ZF-CxxC domain-containing protein lysine-specific demethylase 2A (KDM2A) and its paralogue KDM2B bind to CGIs (12,15–17). KDM2 proteins encode a JmjC domain that catalyses the removal of H3K36 mono- and di- methylation (H3K36me1/2) (18–22). H3K36me1/2 are broadly distributed throughout the mammalian genome (23–25) and H3K36me2 has been proposed to counteract transcription initiation. For example, in yeast H3K36me2 inhibits inappropriate initiation of transcription from cryptic promoters in genes (26–30), and this function may also be conserved in mammals (31,32). Given the seemingly widespread and indiscriminate deposition of H3K36me1/2 in mammalian genomes and its association with transcriptional repression, the discovery that KDM2 proteins localize specifically to CGIs has led to the suggestion that the removal of H3K36me2 at these sites may contribute to a widespread and transcriptionally permissive chromatin state at gene promoters (8,9). Depletion of KDM2A was shown to cause an increase in H3K36me2 at a number of CGI-associated gene promoters, suggesting that KDM2A plays an active role in H3K36me2 removal (12). However, the histone demethylase activity of KDM2 proteins has also been linked to gene repression, including of genes that have roles in cell proliferation, differentiation and senescence (19,33–37). Therefore, the role that KDM2 proteins play in regulating H3K36me1/2 and the effect that this has on CGI-associated gene transcription remain unclear.

**Figure 1.**
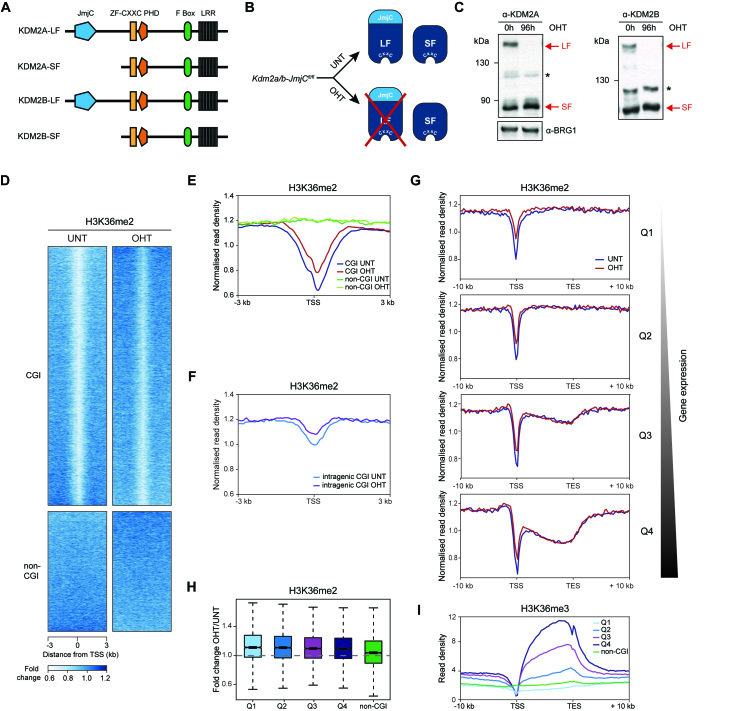
KDM2 proteins contribute modestly to the H3K36me2-depleted state at CGI-associated gene promoters. (**A**) A schematic illustrating protein domain architecture for KDM2A/B long (LF) and short forms (SF). (**B**) A schematic of the *Kdm2a/b-JmjC^fl/fl^* system in which addition of tamoxifen (OHT) leads to removal of KDM2 long forms. (**C**) Western blot analysis for KDM2A and KDM2B in K*dm2a/b-JmjC^fl/fl^* mESCs before (UNT) and after 96 h of tamoxifen (OHT) treatment. BRG1 is shown as a loading control for both blots. Asterisks indicate non-specific bands. (**D**) Heatmaps of H3K36me2 enrichment (ChIP-seq) in K*dm2a/b-JmjC^fl/fl^* mESCs before (UNT) and after addition of tamoxifen (OHT), for CGI-associated (*n* = 14106) and non-CGI-associated (*n* = 6527) gene promoters. H3K36me2 signal was normalized to H3 ChIP-seq to control for any alterations in nucleosome density. (**E**) A metaplot of normalized H3K36me2 ChIP-seq signal at CGI-associated or non-CGI-associated gene promoters in K*dm2a/b-JmjC^fl/fl^* mESCs, before (UNT) and after tamoxifen treatment (OHT). (**F**) A metaplot of normalized H3K36me2 ChIP-seq signal at intragenic CGIs in K*dm2a/b-JmjC^fl/fl^* mESCs, before (UNT) and after tamoxifen treatment (OHT). (**G**) Metaplots showing normalized H3K36me2 ChIP-seq signal throughout the gene body before (UNT) and after tamoxifen treatment (OHT), for CGI-associated genes separated into quartiles according to their expression level in K*dm2a/b-JmjC^fl/fl^* mESCs (Q1 < Q2 < Q3 < Q4). Genes were scaled to the same length and aligned at their TSS and TES. (**H**) A boxplot showing fold change in normalized H3K36me2 ChIP-seq signal following tamoxifen treatment, for the CGI-associated gene quartiles shown in (G) and for non-CGI-associated genes. (**I**) A metaplot showing H3K36me3 enrichment throughout the gene body for the gene sets shown in (G) (62).

KDM2 proteins may also regulate transcription through mechanisms that do not rely on their demethylase activity. KDM2A has been implicated in the formation of pericentromeric heterochromatin (38), whilst KDM2B physically associates with polycomb repressive complex 1 (PRC1) and is required for the formation of repressive polycomb chromatin domains at a subset of CGI-associated gene promoters (15–17,22,39–41). The possibility that KDM2 proteins have demethylase-independent activity is supported by the observation that the *Kdm2a* and *Kdm2b* genes encode internal transcription start sites (TSS) downstream of their JmjC domain. Transcription initiating from these alternative promoters gives rise to short forms of KDM2A and KDM2B (KDM2A/B-SF, Figure 1A) that lack the JmjC domain and therefore cannot act as histone demethylases (14,37). Importantly, however, they retain their ZF-CxxC domain and CGI binding activity. The function of KDM2A/B-SF proteins remains poorly defined, but there is evidence that the KDM2B-SF is sufficient to recruit PRC1 to chromatin (39). Following the depletion of either KDM2A or KDM2B, alterations in gene expression have been reported (12,15,17,39,42). However, whether these two closely related paralogues function cooperatively to regulate gene expression is unknown and, like many chromatin modifying enzymes, it remains largely untested whether they rely on their enzymatic activity for gene regulation. Perhaps more fundamentally, whether the KDM2 proteins function primarily to potentiate or repress gene transcription has not been examined at the genome-scale and remains a major conceptual barrier in understanding how CGIs, which are associated with most vertebrate gene promoters, control gene transcription and expression.

To address these fundamental questions, here we have exploited systematic conditional genetic ablation strategies and detailed genome-wide analysis to dissect how KDM2 proteins regulate H3K36me2 and gene expression in mouse embryonic stem cells (mESCs). We discover that KDM2 proteins contribute only modestly to the H3K36me2-depleted state at CGI-associated gene promoters and the demethylase activity of KDM2 proteins is largely dispensable for normal gene expression. In contrast, surgical removal of the KDM2 ZF-CxxC domains, which liberates KDM2 proteins from CGIs, revealed a widespread increase in gene expression. This was not limited to the function of KDM2B in polycomb-mediated gene repression, but instead occurred broadly across CGI-associated genes, revealing an unexpectedly widespread role for KDM2 proteins in constraining gene expression. KDM2B plays the predominant role in gene repression, whilst KDM2A appears to cooperate with KDM2B to counteract expression at a subset of genes. KDM2-dependent effects on gene expression do not manifest through altered DNA accessibility at CGIs, but instead appear to regulate RNAPII occupancy at gene promoters. Therefore, we define a new demethylase-independent role for KDM2A/B in transcriptional repression, uncovering a new logic whereby CGIs appear, unexpectedly, to constrain gene expression.

## MATERIALS AND METHODS

### Cell culture

Mouse embryonic stem cells (mESCs) were cultured on gelatine-coated dishes at 37°C and 5% CO_2_, in Dulbecco's modified Eagle's medium (DMEM) (Life Technologies) supplemented with 15% fetal bovine serum (Labtech), 2mM L-glutamine (Life Technologies), 0.5 mM beta-mercaptoethanol (Life Technologies), 1× non-essential amino acids (Life Technologies), 1x penicillin-streptomycin (Life Technologies) and 10 ng/ml leukemia-inhibitory factor. Conditional mESC lines were treated with 800 nM 4-hydroxytamoxifen (Sigma) for 96 h to induce KDM2-LFs knockout (*Kdm2a/b-JmjC^fl/fl^*) or ZF-CxxC domain deletion (*Kdm2a/b-CXXC^fl/fl^, Kdm2a-CXXC^fl/fl^, Kdm2b-CXXC^fl/fl^*). For embryoid body differentiation, 10^6^ mESCs were plated on non-adhesive 10 cm dishes in EC-10 medium and cultured for the indicated number of days.

Human HEK293T cells used for KDM2A/B and RNAPII cChIP-seq were grown at 37°C and 5% CO_2_ in DMEM supplemented with 10% fetal bovine serum, 2 mM L-glutamine, 0.5 mM beta-mercaptoethanol and 1× penicillin-streptomycin. *Drosophila melanogaster* S2 (SG4) cells used for cnRNA-seq were grown adhesively at 25°C in Schneider's Drosophila Medium (Life Technologies), supplemented with 1× penicillin-streptomycin and 10% heat-inactivated fetal bovine serum.

### Generation of the *Kdm2a/b-JmjC^fl/fl^* mESC line

To generate the *Kdm2a/b-JmjC^fl/fl^* mESC line, a loxP site was inserted upstream of the critical JmjC domain-encoding exon(s) in the *Kdm2a* and *Kdm2b* genes (exon 8 for *Kdm2a*, and exons 7–8 for *Kdm2b*), and FRT flanked PGK-neo and a second loxP site were inserted downstream of the critical exon(s). Targeting vectors were generated from bacterial artificial chromosomes containing the target mouse genomic regions using the Double Red recombination method, as previously described (43). Linearized targeting vectors were introduced into M1 mESCs by electroporation (GenePulser, Bio-Rad). mESC colonies were isolated and expanded, and the genomic DNA of each clone was purified. Homozygous loxP targeting was verified by sequencing of the genomic region surrounding the loxP sites. Targeted mESCs were injected into mouse blastocysts to generate chimeric mice. The *Kdm2a/b-JmjC^fl/fl^* line was generated by removal of the PGK-neo marker gene by mating the targeted mice with mice expressing FLP recombinase. These *Kdm2a/b-JmjC^fl/fl^* mice were further mated with mice harboring the ROSA26-CreErt2 locus to generate *Kdm2a/b-JmjC^fl/fl^:ROSA26-CreErt2^+/−^* mice, from which the *Kdm2a/b-JmjC^fl/fl^* mESCs used in this study were derived.

### Generation of *Kdm2a-CXXC^fl/fl^* and *Kdm2a/b-CXXC^fl/fl^* mESC lines

Conditional *Kdm2a-CXXC^fl/fl^* and *Kdm2a/b-CXXC^fl/fl^* mESC lines were generated by using CRISPR-mediated genome editing to insert parallel loxP sites flanking exon 14 of the *Kdm2a* gene in *Rosa26::CreERT2* or *Kdm2b-CXXC^fl/fl^* mESCs, respectively (39). Targeting constructs encoding the loxP sequence flanked by 150 bp homology arms and carrying a mutated PAM sequence to prevent retargeting by the Cas9 enzyme were purchased from GeneArt (ThermoFisher). The pSpCas9(BB)-2A-Puro(PX459)-V2.0 vector was obtained from Addgene (#62988). sgRNAs were designed using the CRISPOR online tool (http://crispor.tefor.net/crispor.py) and were cloned into the vector as previously described (44). First, the upstream loxP site was targeted. *Rosa26::CreERT2* or *Kdm2b-CXXC^fl/fl^* mESCs were transiently co-transfected with 1 μg of Cas9-sgRNA plasmid and 3.5 μg of targeting construct using Lipofectamine 3000 (ThermoFisher). The day after transfection, cells were passaged at a range of densities and subjected to puromycin selection (1 μg/ml) for 48 h. Individual clones were isolated and screened by polymerase chain reaction (PCR). A correctly targeted homozygous clone was then used to target the downstream loxP site using the same transfection protocol and screening strategy. Correct loxP targeting was verified by sequencing of the genomic region surround the loxP sites, and clones were analysed by both RT-qPCR and western blot to confirm loss of the ZF-CxxC domain in response to tamoxifen treatment.

### Protein extracts and immunoblotting

For nuclear extraction, mESCs were washed with phosphate-buffered saline (PBS) then resuspended in 10 volumes of Buffer A (10 mM Hepes pH 7.9, 1.5 mM MgCl_2_, 10 mM KCl, 0.5 mM DL-dithiothreitol (DTT), 0.5 mM phenylmethylsulfonyl fluoride (PMSF) and 1× PIC (Roche)) and incubated on ice for 10 min. Cells were recovered by centrifugation at 1500 *g* for 5 min, resuspended in three volumes of Buffer A supplemented with 0.1% NP-40 and incubated on ice for 10 min. The released nuclei were recovered by centrifugation at 1500 *g* for 5 min and resuspended in 1 pellet volume of Buffer B (5 mM Hepes pH 7.9, 26% glycerol, 400 mM NaCl, 1.5 mM MgCl_2_, 0.2 mM ethylenediaminetetraacetic acid (EDTA), 0.5 mM DTT and 1× PIC). After 1 h of rotation at 4°C, the suspension was pelleted at 16 000 *g* for 20 min and the supernatant taken as nuclear extract.

For histone extraction, mESCs were washed with RSB (10 mM Tris–HCl pH 7.4, 10 mM NaCl, 3 mM MgCl_2_ and 20 mM NEM), then resuspended in RSB buffer supplemented with 0.5% NP-40 and incubated on ice for 10 min to allow cell lysis. Following centrifugation at 500 *g* for 5 min, the nuclear pellet was incubated in 2.5 mM MgCl_2_, 0.4M HCl and 20 mM NEM on ice for 20 min. After centrifugation at 16 000 *g* for 20 min, histones were precipitated from the supernatant on ice with 25% TCA for 30 min. Histones were recovered by centrifugation at 16 000 *g* for 15 min, and the pellet was washed twice in acetone. The histone pellet was resuspended in 1× sodium dodecyl sulphate (SDS) loading buffer and boiled at 95°C for 5 min. Any insoluble precipitate was pelleted by centrifugation at 16 000 *g* for 15 min and the soluble fraction retained as histone extract. Histone concentrations were compared by Coomassie Blue staining following SDS-polyacrylamide gel electrophoresis. Semi-quantitative western blot analysis of histone extracts was performed using LiCOR IRDye^®^ secondary antibodies and the LiCOR Odyssey Fc system. To measure changes in H3K36 methylation, the signal relative to histone H4 was determined.

### Antibodies

The following antibodies were used in this study: anti-KDM2A (12), anti-KDM2B (15), anti-BRG1 (EPNCIR111A, Abcam), anti-H3, anti-H3K36me1, anti-H3K36me2 (12), anti-H3K36me3, anti-H4 (L64C1, Cell Signalling), anti-H2AK119ub1 (D27C4, Cell Signalling), anti-H3K27me3 (45), anti-Rbp1-NTD (D8L4Y, Cell Signalling), anti-Rbp1-CTD-Ser5P (D9N5I, Cell Signalling), anti-Rbp1-CTD-Ser2P (E1Z3G, Cell Signalling). Anti-H3 and anti-H3K36me antibodies were prepared in-house by rabbit immunization with synthetic peptides (PTU/BS Scottish National Blood Transfusion Service) and antibodies were purified on peptide affinity columns.

### Preparation of chromatin

For histone H3 and H3K36me2 ChIP, 1 × 10^7^ mESCs were crosslinked for 10 min in 1% formaldehyde. Reactions were quenched by addition of 125 mM glycine. The released nuclei were washed twice in PBS, then resuspended in lysis buffer (1% SDS, 10 mM EDTA, 50 mM Tris–HCl pH 8.1 and 1 × PIC) and incubated on ice for 30 min.

For KDM2A/B ChIP, 5 × 10^7^ mESCs were resuspended in PBS and mixed with 2 × 10^6^ HEK293T cells. Cells were crosslinked in 2 mM disuccinimidyl glutarate (Thermo Scientific) for 45 min at 25°C with gentle rotation, then in 1% formaldehyde for 12.5 min (methanol-free, Life Technologies). Reactions were quenched by addition of 125 mM glycine, and crosslinked cells were resuspended in lysis buffer (50 mM HEPES-KOH pH 7.9, 140 mM NaCl, 1 mM EDTA, 10% glycerol, 0.5% NP40, 0.25% TritonX-100 and 1× PIC) and rotated for 10 min at 4°C. The released nuclei were washed (10 mM Tris–HCl pH 8.0, 200 mM NaCl, 1 mM EDTA, 0.5 mM EGTA and 1× PIC) for 5 min at 4°C, and the nuclear pellet resuspended in 1 ml sonication buffer (10 mM Tris–HCl pH 8.0, 100 mM NaCl, 1 mM EDTA, 0.5 mM EGTA, 0.1% sodium deoxycholate, 0.5% N-lauroylsarcosine and 1× PIC).

For RNAPII ChIP, 5 × 10^7^ mESCs were resuspended in PBS and mixed with 4 × 10^6^ HEK293T cells. Cells were crosslinked for 10 min in 1% formaldehyde. Reactions were quenched by addition of 150 mM glycine and the crosslinked cells resuspended in FA-lysis buffer for 10 min (50 mM HEPES pH 7.9, 150 mM NaCl, 2 mM EDTA, 0.5 mM EGTA, 0.5% NP40, 0.1% sodium deoxycholate, 0.1% SDS, 10 mM NaF, 1 mM AEBSF, 1×PIC).

Chromatin was sonicated using a BioRuptor Pico sonicator (Diagenode), shearing genomic DNA to ∼0.5 kb. Sonicated chromatin was diluted 10-fold in ChIP dilution buffer (1% TritonX-100, 1 mM EDTA, 20 mM Tris–HCl pH 8, 150 mM NaCl and 1× PIC) for H3, H3K36me2 or KDM2A/B ChIP, or in FA-lysis buffer for RNAPII ChIP.

For H2AK119ub1 and H3K27me3 ChIP, native chromatin was prepared as previously described (46).

### Chromatin immunoprecipitation and sequencing

For fixed ChIP, chromatin was pre-cleared for 1 h with either protein A agarose beads (Repligen, for H3, H3K36me2 or RNAPII ChIP) or protein A magnetic Dynabeads (Invitrogen, for KDM2A/B ChIP) blocked with 1 mg/ml bovine serum albumin (BSA) and 1 mg/ml yeast tRNA. For each ChIP reaction, 150 μg chromatin (KDM2A/B), 300 μg chromatin (RNAPII) or chromatin corresponding to 1 × 10^5^ cells (H3 or H3K36me2 ChIP) was incubated overnight with the appropriate antibody: anti-H3 (15 μl), anti-H3K36me2 (15 μl), anti-KDM2A (1.2 μl), anti-KDM2B (1 μl), anti-Rbp1-NTD (15 μl), anti-Rbp1-CTD-Ser5P (12.5 μl), anti-Rbp1-CTD-Ser2P (12.5 μl).

Antibody-bound chromatin was isolated using blocked protein A agarose (H3, H3K63me2 or RNAPII ChIP) or magnetic beads (KDM2A/B ChIP) for 2 h at 4°C. For H3, H3K36me2 or KDM2A/B ChIP, washes were performed with low salt buffer (0.1% SDS, 1% TritonX-100, 2 mM EDTA, 20 mM Tris–HCl pH 8, 150 mM NaCl), high salt buffer (0.1% SDS, 1% TritonX-100, 2 mM EDTA, 20 mM Tris–HCl pH 8, 500 mM NaCl), LiCl buffer (250 mM LiCl, 1% NP40, 1% sodium deoxycholate, 1 mM EDTA, 10 mM Tris–HCl pH 8) and two washes with TE buffer (10 mM Tris–HCl pH 8, 1 mM EDTA). For RNAPII ChIP, washes were performed with FA-Lysis buffer, FA-Lysis buffer containing 500 mM NaCl, DOC buffer (250 mM LiCl, 0.5% NP40, 0.5% sodium deoxycholate, 2 mM EDTA, 10 mM Tris–HCl pH 8) and two washes with TE buffer. ChIP DNA was eluted in elution buffer (1% SDS, 100 mM NaHCO_3_) and crosslinks reversed overnight at 65°C with 200 mM NaCl and 2 μl RNase A (Sigma). A matched input sample (corresponding to 10% of original ChIP reaction) was treated identically. The following day, samples were treated with 20 μg/ml Proteinase K (Sigma) for 2 h at 45°C and purified using the ChIP DNA Clean and Concentrator Kit (Zymo Research).

For H2AK119ub1 or H3K27me3 ChIP, S1/S2 nucleosomes were diluted 10-fold in native ChIP incubation buffer (70 mM NaCl, 10 mM Tris–HCl pH 7.5, 2 mM MgCl_2_, 2 mM EDTA, 0.1% TritonX-100, 10 mM NEM, 1× PIC). For each ChIP reaction, 1 ml of diluted nucleosomes was incubated overnight at 4°C with 5 μl of anti-H2AK119ub1 or anti-H3K27me3 antibody. Antibody-bound nucleosomes were isolated by incubation for 1 h at 4°C with protein A agarose beads, which were pre-blocked overnight in native ChIP incubation buffer supplemented with 1 mg/ml BSA and 1 mg/ml yeast tRNA. The beads were then washed four times with native wash buffer (20 mM Tris–HCl pH 7.5, 2 mM EDTA, 125 mM NaCl, 0.1% TritonX-100) and once with TE buffer. ChIP DNA was eluted using 100 μl of elution buffer (1% SDS, 0.1 M NaHCO_3_), then purified using the ChIP DNA Clean and Concentrator Kit (Zymo Research). DNA from a matched input sample (corresponding to 10% of original ChIP reaction) was also purified.

ChIP-seq libraries for both ChIP and input samples were prepared using the NEBNext Ultra DNA Library Prep Kit for Illumina (NEB), following the manufacturer's guidelines and using NEBNext Multiplex Oligos. The average size and concentration of libraries were determined using the 2100 Bioanalyzer High Sensitivity DNA Kit (Agilent) and qPCR with SensiMix SYBR (Bioline) and KAPA Illumina DNA standards (Roche). Libraries were sequenced using the Illumina NextSeq 500 platform in biological triplicate or quadruplicate with 40 bp paired-end reads.

### Calibrated nuclear RNA-sequencing and ATAC-sequencing (cnRNA-seq and cATAC-seq)

To isolate nuclei for cnRNA-seq and cATAC-seq, 1 × 10^7^ mESCs were mixed with 2.5 × 10^6^*Drosophila* SG4 cells in PBS. Cells were lysed in 1 ml HS lysis buffer (0.05% NP40, 50 mM KCl, 10 mM MgSO_4_.7H_2_0, 5 mM HEPES, 1 mM PMSF, 3 mM DTT, 1× PIC). Nuclei were recovered by centrifugation at 1000 *g* for 5 min and washed three times in 1 ml resuspension buffer (10 mM NaCl, 10 mM Tris–HCl pH 7.4, 3 mM MgCl_2_). Nuclear integrity was assessed using 0.4% trypan blue staining (ThermoFisher).

Nuclear RNA was prepared from 4 × 10^6^ nuclei using TRIzol reagent according to the manufacturer's protocol (Invitrogen), then treated with the TURBO DNA-free kit (ThermoFisher). nRNA quality was assessed using the 2100 Bioanalyzer RNA 6000 Pico kit (Agilent), then nRNA was depleted of rRNA using the NEBNext rRNA depletion kit and the depletion efficiency evaluated using the Bioanlayzer RNA 6000 Pico kit. RNA-seq libraries were prepared using the NEBNext Ultra Directional RNA-seq kit, and library size and concentration was determined as described for ChIP libraries. Libraries were sequenced using the Illumina NextSeq 500 platform in biological triplicate or quadruplicate using 80 bp paired-end reads.

Chromatin accessibility was assayed using an adaptation of the assay for transposase accessible-chromatin (ATAC)-seq (47) as previously described (48), using 5 × 10^5^ nuclei from the same preparation used for the purification of nuclear RNA. Genomic DNA was also purified from an aliquot of the same preparation of nuclei by phenol-chloroform extraction and tagmented with Tn5, to control for sequence bias of the Tn5 transposase and to determine the exact mouse/fly mixing ratio for each individual sample.

ATAC-seq and input gDNA libraries were prepared by PCR amplification using custom-made Illumina barcodes (47) and the NEBNext High-Fidelity 2X PCR Master Mix. Libraries were purified with two rounds of Agencourt AMPure XP bead cleanup (Agencourt, 1.5× bead:sample ratio). Library size and concentration were determined as described for ChIP libraries. Libraries were sequenced using the Illumina NextSeq 500 platform in biological triplicate or quadruplicate using 80 bp paired-end reads.

### Droplet digital PCR

For droplet digital PCR (ddPCR), total RNA was prepared from 10^6^ mESCs using the RNeasy mini plus kit including gDNA eliminator columns (QIAGEN). RPT low retention tips (Starlab) were used throughout the ddPCR protocol to increase pipetting accuracy. Purified RNA was eluted in 30 μl elution buffer, 7 μl was diluted with 8 μl water and 4 μl of this dilution was reverse transcribed using the imProm-II system with random hexamer primers and RNasin ribonuclease inhibitor (Promega). The generated cDNA was diluted with 300 μl nuclease-free water. ddPCR primers were designed using Primer 3 Plus (49) with BioRad recommended settings: 3.8 mM divalent cations, 0.8 mM dNTPs, 80–120 bp product, 60–61°C melting temperature. Their efficiency was tested using a serial dilution curve of cDNA by standard SYBR qPCR. ddPCR reactions were prepared in 96-well PCR plates, and contained 12.5 μl 2× QX200 ddPCR EvaGreen Supermix (BioRad), 0.32 μl each of forward and reverse primers (10 μM), 7 μl diluted cDNA and 4.86 μl nuclease-free water. This 25 μl reaction was mixed by pipetting, then 22 μl was transferred to a semi-skirted 96-well PCR plate (Eppendorf) and used for droplet generation with an AutoDG droplet generator (BioRad). Droplets were collected in a semi-skirted PCR plate, which was then sealed using a PX1 PCR plate sealer (BioRad). PCR was performed using a C1000 Touch thermal cycler (BioRad) with a 2°C/s ramp rate: 5 min at 95°C followed by 40 cycles of denaturation at 95°C for 30 s and annealing/extension at 60°C for 60 s, then signal stabilization at 4°C for 5 min followed by 90°C for 5 min. Droplets were sorted into PCR-positive and PCR-negative fractions according to their fluorescence using the QX200 droplet reader (BioRad). QuantaLife software (BioRad) was used to calculate the absolute concentration of template cDNA in the ddPCR reaction.

### Data processing and normalization of massively parallel sequencing

For cATAC-seq and KDM2A, KDM2B and RNAPII cChIP-seq (including sequencing of input gDNA), reads were aligned to concatenated mouse and spike-in genomes (mm10+dm6 or mm10+hg19) using Bowtie 2 with the ‘– no-mixed’ and ‘– no-discordant’ options (50). For histone ChIP-seq, reads were aligned to the mouse mm10 genome as above. Reads that were mapped more than once were discarded, and PCR duplicates were removed using SAMTools (51). For cATAC-seq, reads that mapped to a custom ‘blacklist’ of genomic regions with artificially high counts, including mitochondrial DNA sequences, were also discarded.

For cnRNA-seq, reads were first aligned using Bowtie 2 (with ‘–very-fast’, ‘–no-mixed’ and ‘–no-discordant’ options) to the concatenated mm10 and dm6 rRNA genomic sequence (GenBank: BK000964.3 and M21017.1) to filter out reads mapping to rRNA. All unmapped reads were aligned to the concatenated mm10+dm6 genome using STAR (52). To improve mapping of intronic sequences, reads that failed to map using STAR were aligned using Bowtie 2, with ‘–sensitive-local’, ‘–no-mixed’ and ‘–no-discordant’ options. PCR duplicates were removed using SAMtools (51).

To internally calibrate cnRNA-seq, cATAC-seq and cChIP-seq experiments we spiked a fixed number of control cells into each sample (*Drosophila* SG4 cells for cnRNA-seq and cATAC-seq, human HEK293T cells for KDM2A/B and RNAPII cChIP-seq). This spike-in genome was then used to quantitatively compare the gene expression, chromatin accessibility or ChIP-seq profiles between experimental conditions. For visualization of cATAC-seq and cChIP-seq data, mm10 reads were randomly subsampled by a factor that reflects the total number of spike-in reads in the same sample, as previously described (53–55). To account for any variation in the exact spike-in cell: mESC mixing ratio between biological replicates, the subsampling factors were additionally corrected according to the ratio of dm6 (or hg19)/mm10 total read counts in the matched input sample. For visualization of cnRNA-seq data, mm10 reads were randomly subsampled by *Drosophila* normalized size factors calculated using DESeq2 (see below). Histone ChIP-seq libraries were randomly downsampled to achieve the same total number of reads for each individual replicate using SAMtools (51).

### Read count quantitation and analysis

To compare replicates, read coverage across regions of interest (gene bodies for cnRNA-seq and ChIP-seq, gene promoters for cATAC-seq) was analysed using deepTools multiBamSummary and plotCorrelation functions (56). For each condition, biological replicates correlated well with each other (Pearson correlation coefficient > 0.95) and were merged for downstream applications.

Genome coverage tracks were generated using the pileup function from MACS2 (57) for ChIP-seq and ATAC-seq and genomeCoverageBed from BEDtools (58) for cnRNA-seq and visualized using the UCSC genome browser (59). Differential bigwig tracks of H3K36me2 normalized to H3 or of RNAPII signal in tamoxifen-treated versus –untreated cells were generated from merged bigwig files using the deepTools bigwigCompare function with ‘–operation ratio’ setting (56). Metaplot and heatmap analyses of read density were performed using the computeMatrix, plotProfile and plotHeatmap deepTools functions (v3.0.1). For ChIP-seq, intervals of interest were annotated with normalized read counts from merged replicates with a custom Perl script using SAMtools, or from differential bigwig files using deepTools computeMatrix with the ‘–outFileNameMatrix’ option. Correlation analyses were performed in R using Spearman correlation and visualized with scatterplots coloured by density using ‘stat_density2d’.

### Differential ATAC-seq and gene expression analyses

DESeq2 (60) was used with a custom R script to identify significant changes in chromatin accessibility or gene expression. In order to calibrate to the spike-in genome, *Drosophila* reads were first pre-normalized according to the exact dm6/mm10 spike-in ratio derived from the matched input gDNA sample. *Drosophila* read counts were then generated for a set of unique dm6 refGene genes and used to calculate DESeq2 size factors. These size factors were supplied for DESeq2 normalization of raw mm10 read counts for a custom non-redundant mm10 gene set of 20633 genes. P-adj < 0.05 and fold change > 1.4 thresholds were used to determine significant changes. Log2 fold changes were visualized using MA-plots generated with ggplot2.

### Gene annotation

Non-redundant mouse genes (*n* = 20633) were classified into non-CGI, PRC and non-PRC categories based on the presence of a non-methylated CGI and RING1B and SUZ12 binding at their promoters. Gene Ontology analysis was performed using DAVID (61). The BP FAT setting and an FDR < 0.1 cut-off were used, and the complete non-redundant mm10 gene set was used as a background.

### Accession numbers

The following previously published datasets were used for analysis: H3K36me3 ChIP-seq (GSE34520) (62), BioCAP (GSE43512) (63), *Pcgf1^fl/fl^* cnRNA-seq, RING1B ChIP-seq and SUZ12 ChIP-seq (GSE119620) (46).

## RESULTS

### KDM2 proteins contribute modestly to the H3K36me2-depleted state at CGI-associated gene promoters

KDM2A and KDM2B both catalyse H3K36me2 demethylation via their JmjC domain (19,20) and localize to CGIs via their ZF-CxxC domain (Figure 1A) (12,15–17). However, whether KDM2 proteins regulate H3K36me2 at CGI-associated gene promoters throughout the genome has not been examined. Therefore, we generated a mESC system in which loxP sites were inserted into the *Kdm2a* and *Kdm2b* genes flanking exons that encode the JmjC domain (*Kdm2a/b-JmjC^fl/fl^*, Supplementary Figure S1A) and which also expresses a tamoxifen-inducible form of CRE recombinase. Addition of tamoxifen triggers deletion of the JmjC domain-containing exons, removing the long forms of KDM2A and KDM2B (KDM2-LFs) and their associated demethylase activity (Figure 1B and C; Supplementary Figure S1B). Importantly, KDM2-SFs, which are expressed from downstream promoters (Supplementary Figure S1A), were unaffected by removal of the KDM2-LFs (Figure 1 B and C). We first investigated the contribution of KDM2-LFs to global H3K36 methylation levels by western blot, and observed only minor changes following removal of KDM2-LFs (Supplementary Figure S1C and D). Next, we examined the genome-wide distribution of H3K36me2 using chromatin immunoprecipitation followed by massively parallel sequencing (ChIP-seq). This confirmed a local depletion of H3K36me2 at CGI-associated gene promoters (Figure 1D and E; Supplementary Figure S1E) (8,9,12). H3K36me2 depletion was not detected at non-CGI gene promoters, demonstrating that this is a CGI-associated chromatin feature. Following removal of the KDM2-LFs by tamoxifen treatment, there was a modest increase in H3K36me2 at the TSS of CGI-associated gene promoters (Figure 1D and E; Supplementary Figure S1E) which we also validated by ChIP-qPCR (Supplementary Figure S1F). This demonstrates that KDM2A/B contribute to the H3K36me2-depleted state at CGIs, in agreement with single-gene studies examining the KDM2A- or KDM2B-depleted state (12). Interestingly, intragenic CGIs were also depleted of H3K36me2 (Figure 1F), although this depletion was on average less pronounced than at CGI promoters, likely due to their lower average CpG density and size (Supplementary Figure S1G). Importantly, removal of the KDM2-LFs resulted in an increase in H3K36me2 at intragenic CGIs, indicating that their H3K36me2-depleted state is also shaped by KDM2A/B.

Our ChIP-seq analysis revealed that KDM2-LFs contribute to the H3K36me2-depleted state at CGI-associated promoters. However, we were curious whether the effects on H3K36me2 were uniformly distributed or dependent on other features of gene promoters, such as transcriptional activity. Therefore, we separated genes based on expression level (Supplementary Figure S1H) and examined H3K36me2 at genes and surrounding regions. This revealed that CGI-associated TSSs were depleted of H3K36me2 irrespective of expression level (Figure 1G). Chromatin surrounding CGI-associated TSSs was blanketed by H3K36me2, consistent with this modification being pervasive in mammalian genomes. The increase in H3K36me2 at the TSS following KDM2-LF removal was similar across all expression levels (Figure 1H), consistent with the transcription-independent targeting of KDM2 proteins to CGI promoters via their ZF-CxxC domains. Interestingly, highly transcribed genes were also depleted of H3K36me2 in their gene body (Figure 1G). This did not result from an enrichment of short genes in the set of highly transcribed genes (Supplementary Figure S1I), and was independent of KDM2 demethylase activity. Instead this depletion correlated with co-transcriptional conversion of H3K36me2 to H3K36me3 (Figure 1I and Supplementary Figure S1J) (64–69). Together, these observations reveal that KDM2 proteins remove H3K36me2 from CGIs, but unexpectedly their contribution to the depletion of H3K36me2 at these sites is modest. This suggests that CGIs could be inherently refractory to H3K36me2 or that additional H3K36 demethylases may also function at these regions (see Discussion).

### KDM2 demethylase activity contributes minimally to gene regulation

**Figure 2.**
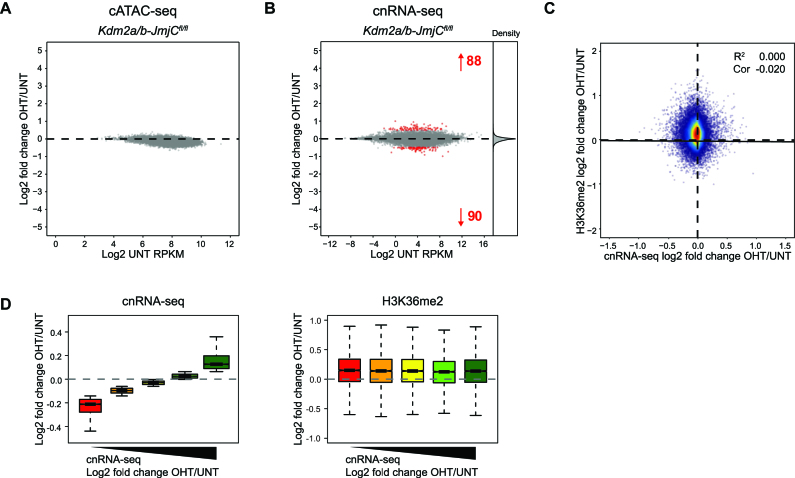
KDM2 demethylase activity contributes minimally to gene regulation. (**A**) An MA-plot showing log2 fold change in the accessibility (cATAC-seq) of CGI-associated gene promoters in K*dm2a/b-JmjC^fl/fl^* mESCs following tamoxifen treatment. No promoters significantly changed in accessibility (p-adj < 0.05 and > 1.4-fold). (**B**) An MA-plot showing log2 fold change in gene expression (cnRNA-seq) in K*dm2a/b-JmjC^fl/fl^* mESCs following tamoxifen treatment. The number of genes with significantly increased or decreased expression (p-adj < 0.05 and > 1.4-fold) is shown in red and density of gene expression changes is shown on the right. (**C**) A scatter plot comparing the log2 fold change in gene expression (cnRNA-seq) with the log2 fold change in normalized H3K36me2 ChIP-seq signal for CGI-associated genes following tamoxifen treatment of *Kdm2a/b-JmjC^fl/fl^* mESCs. The solid line shows the linear regression, and the coefficient of determination (*R*^2^) and Spearman correlation coefficient (Cor) are annotated. (**D**) Boxplots showing the log2 fold change in cnRNA-seq signal (left) and normalized H3K36me2 ChIP-seq signal (right) for CGI-associated genes grouped into quintiles based on their change in expression following tamoxifen treatment of *Kdm2a/b-JmjC^fl/fl^* mESCs.

Depletion of H3K36me2 at CGI-associated gene promoters has been proposed to contribute to the generation of a transcriptionally permissive chromatin state (8,9,12). Although KDM2 proteins appear to contribute only modestly to the H3K36me2-depleted state at CGIs (Figure 1), we were curious whether this effect was nevertheless required to sustain normal chromatin accessibility and transcription from CGI-associated gene promoters. To address these questions, we first performed calibrated ATAC-seq (cATAC-seq) to measure chromatin accessibility before and after removal of KDM2-LFs. This demonstrated that CGI promoters remained accessible, despite the observed increases in H3K36me2 (Figure 2A and Supplementary Figure S2A). To examine gene expression, we performed calibrated nuclear RNA sequencing (cnRNA-seq). This revealed that the expression of the vast majority of genes did not change following removal of KDM2 demethylase activity, with only a small number of genes being modestly perturbed (Figure 2B). Furthermore, there was a poor correlation between gene expression changes and the effects on H3K36me2 at gene promoters (Figure 2C and D). This minimal perturbation to chromatin accessibility and gene expression following loss of KDM2-LFs indicates that histone demethylase activity of KDM2 proteins is largely dispensable for normal CGI-associated promoter activity.

### KDM2 proteins play a widespread role in gene repression

**Figure 3.**
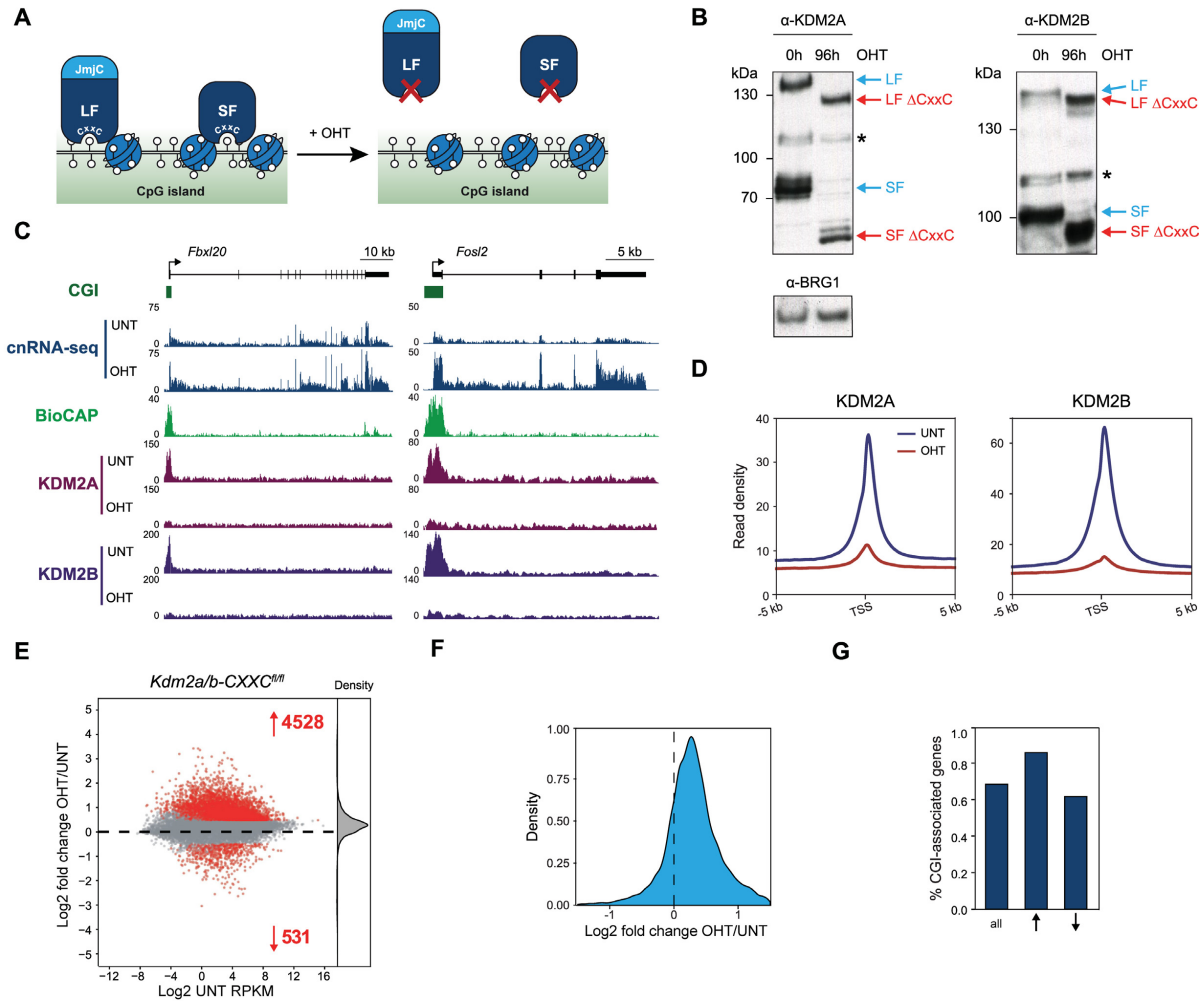
KDM2 proteins mediate widespread gene repression. (**A**) A schematic of the *Kdm2a/b-CXXC^fl/fl^* system in which addition of tamoxifen (OHT) leads to the generation of KDM2 proteins that lack the ZF-CxxC domain and therefore are unable to bind to chromatin. (**B**) Western blot analysis for KDM2A and KDM2B in *Kdm2a/b-CXXC^fl/fl^* mESCs before (UNT) and after 96 h tamoxifen treatment (OHT). BRG1 is shown as a loading control for both blots. Asterisks indicate non-specific bands. (**C**) Genomic snapshots showing gene expression (cnRNA-seq) and KDM2A and KDM2B ChIP-seq before (UNT) and after tamoxifen treatment (OHT) of *Kdm2a/b-CXXC^fl/fl^*mESCs, for representative genes that moderately (*Fbxl20*, left) or more dramatically increased in expression (*Fosl2*, right). BioCAP signal is shown for reference (63). (**D**) Metaplots showing enrichment of KDM2A and KDM2B ChIP-seq signal at the TSS of all CGI-associated genes (*n* = 14106) before (UNT, blue) and after tamoxifen treatment of *Kdm2a/b-CXXC^fl/fl^* mESCs (OHT, red). (**E**) An MA-plot showing log2 fold change in gene expression (cnRNA-seq) in K*dm2a/b-CXXC^fl/fl^* mESCs following tamoxifen treatment. The number of genes with significantly increased or decreased expression (p-adj < 0.05 and > 1.4-fold) are shown in red and density of gene expression changes is shown on the right. (**F**) A density plot showing the distribution of the log2 fold change in gene expression following tamoxifen treatment of *Kdm2a/b-CXXC^fl/fl^* mESCs, for all genes. (**G**) A bar graph showing the proportion of genes that have a CGI promoter, for all genes and genes that significantly increased or decreased in expression following tamoxifen treatment of *Kdm2a/b-CXXC^fl/fl^* mESCs.

Given that gene expression was largely unaffected when KDM2 demethylase activity was removed, we wondered whether demethylase-independent activities of KDM2 proteins may play a more prominent role in gene regulation. KDM2A and KDM2B encode multiple isoforms, each of which contain the ZF-CxxC DNA binding domain. Therefore, to remove all CGI-targeted KDM2 proteins, we developed a conditional mESC system in which the exon encoding the ZF-CxxC domain is flanked by loxP sites in both *Kdm2a* and *Kdm2b* genes (*Kdm2a/b-CXXC^fl/fl^*), and which expresses tamoxifen-inducible CRE recombinase (Supplementary Figure S3A). Following addition of tamoxifen the ZF-CxxC-encoding exons are excised, producing KDM2A and KDM2B proteins that now lack the ZF-CxxC domain (Figure 3A). The effectiveness of this approach was evident from an increased mobility of the KDM2 proteins in western blot analysis (Figure 3B). KDM2 proteins lacking their ZF-CxxC domain no longer bound to CGIs as assessed by ChIP (Figure 3C and D; Supplementary Figure S3B). Furthermore, we observed an increase in H3K36me2 at CGI-associated gene promoters (Supplementary Figure S3C) consistent with both long and short form KDM2 proteins dissociating from chromatin.

To examine whether this loss of CGI binding had an effect on gene expression, we carried out cnRNA-seq and compared gene expression between untreated and tamoxifen-treated cells. This revealed that KDM2 protein removal resulted in more than a fifth of all genes showing significantly increased expression (Figure 3C and E). Owing to the quantitative nature of cnRNA-seq it was also apparent that KDM2 protein removal led to a more general increase in gene expression, even amongst genes that were not considered significantly changed by statistical analysis (Figure 3F). We validated these widespread effects using highly sensitive and quantitative droplet digital PCR analysis (Supplementary Figure S3D). Importantly, our capacity to uncover this broad increase in gene expression was only possible due to the use of calibrated nuclear RNA-seq (cnRNA-seq) as conventional normalization based on total read count fails to uncover this pervasive alteration in gene expression (Supplementary Figure S3E). When we examined in more detail the transcripts with significantly increased expression these were enriched for CGI-associated genes (Figure 3G), consistent with these effects being a direct result of KDM2 protein removal as opposed to a global perturbation of some core transcriptional component. In contrast, significantly downregulated genes were less numerous, not enriched for CGI-associated genes and encompassed both polycomb-bound and unbound genes (Supplementary Figure S3F), suggesting they may correspond to secondary effects. Together, these observations establish an unexpected and widespread role for KDM2 proteins in suppressing expression from CGI-associated gene promoters.

### Elevated gene expression following KDM2 protein removal is not simply a consequence of polycomb target gene reactivation

**Figure 4.**
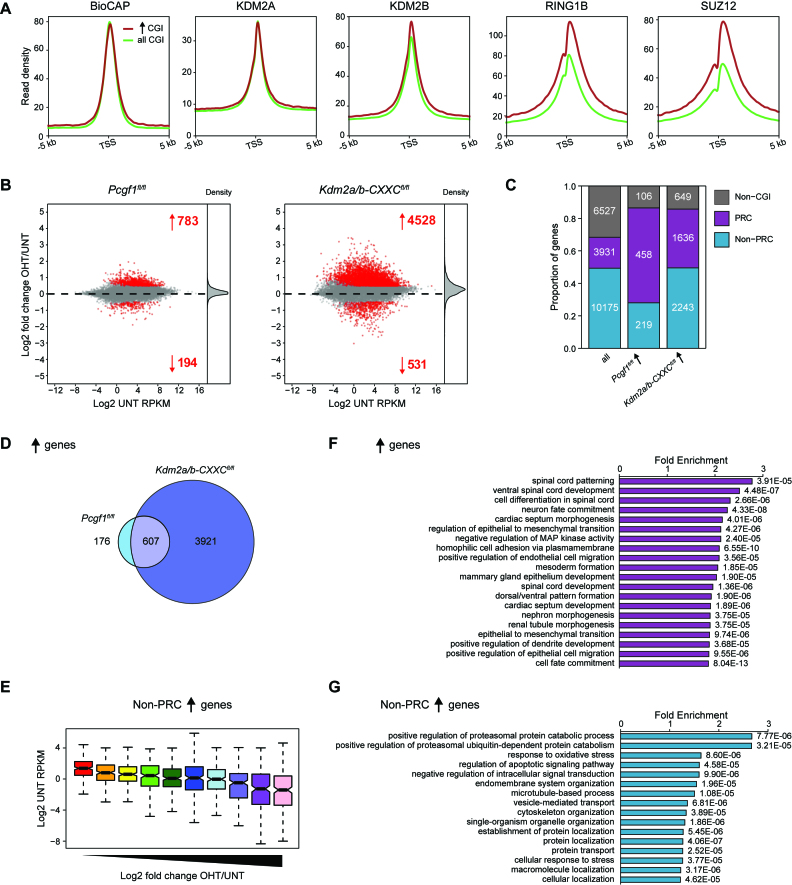
KDM2-mediated repression is not limited to polycomb target genes. (**A**) Metaplots showing enrichment of BioCAP signal and KDM2A, KDM2B, RING1B and SUZ12 ChIP-seq signal at the TSS of all CGI-associated genes (*n* = 14106, green) and of the subset of these genes that significantly increased in expression following tamoxifen treatment of *Kdm2a/b-CXXC^fl/fl^* mESCs (*n* = 3879, red) (46,63). (**B**) Left: an MA-plot showing log2 fold change in gene expression (cnRNA-seq) in *Pcgf1^fl/fl^* mESCs following tamoxifen treatment to induce PCGF1 knockout (46). The number of genes with significantly increased or decreased expression (p-adj < 0.05 and > 1.4-fold) is shown in red and density of gene expression changes is shown on the right. Right: as left but for *Kdm2a/b-CXXC^fl/fl^* mESCs, shown for comparison. (**C**) A bar graph comparing the distribution of genes into three classes—non-CGI, polycomb (PRC) occupied and non-PRC occupied—for all genes and for genes that significantly increased in expression following tamoxifen treatment of *Pcgf1^fl/fl^* or *Kdm2a/b-CXXC^fl/fl^* mESCs. Non-CGI genes are genes that lack a CGI at their promoter. Non-PRC-occupied genes have a CGI promoter that is not bound by polycomb complexes, whilst PRC-occupied genes have a CGI promoter that is bound by polycomb complexes. (**D**) A Venn diagram showing the overlap between genes that significantly increased in expression following tamoxifen treatment of *Pcgf1^fl/fl^* and *Kdm2a/b-CXXC^fl/fl^* mESCs. (**E**) A box plot showing the starting expression level (log2 UNT RPKM) for genes grouped into deciles based on their log2 fold change in expression following tamoxifen treatment of *Kdm2a/b-CXXC^fl/fl^* mESCs. (**F**) Gene ontology analysis of genes that significantly increased in expression following tamoxifen treatment of *Kdm2a/b-CXXC^fl/fl^* mESCs. (**G**) As (**H**), but for the subset of significantly increasing genes that were not classified as polycomb target genes.

We have previously shown that KDM2B plays an important role in recruiting the polycomb repressive complex 1 (PRC1) to CGI-associated gene promoters in mESCs. KDM2B does so by binding to an adaptor protein, called PCGF1, which directly interacts with RING1B, the catalytic core of PRC1 (15–17,39). PRC1 can ubiquitylate histone H2A at position 119 (H2AK119ub1), which then stabilizes the second polycomb repressive complex, PRC2, which can trimethylate histone H3 lysine 27 (H3K27me3). When we examined the genes that increased in expression following KDM2 protein removal they had stereotypical CGI-associated features (Figure 4A), but interestingly were also enriched for RING1B, a core structural component of PRC2 (SUZ12), and had H2AK119ub1 and H3K27me3 (Supplementary Figure S4A). In agreement with KDM2B having the capacity to target the polycomb system to CGI-associated genes, H2AK119ub1 and H3K27me3 were reduced at CGI-associated TSSs in tamoxifen-treated *Kdm2a/b-CXXC^fl/fl^* cells (Supplementary Figure S4B–D). This effect was not evident in *Kdm2a/b-JmjC^fl/fl^ cells*, in keeping with our previous observations that the KDM2B SF is sufficient to target PRC1 (39) (Supplementary Figure S4E–G). Furthermore, removal of PCGF1, the adaptor protein that links KDM2B to PRC1, caused similar reductions in polycomb-associated histone modifications (46). The observed effects on polycomb-mediated histone modifications following tamoxifen treatment of *Kdm2a/b-CXXC^fl/fl^* cells raised the possibility that the widespread increase in gene expression following KDM2 protein removal may simply result from loss of KDM2B-dependent targeting and gene repression by the PRC1 complex. To investigate this possibility, we compared the gene expression changes following KDM2 protein removal with those following conditional removal of PCGF1 (46). This revealed that removal of PCGF1 caused de-repression of more than four times fewer genes than removal of KDM2 proteins (Figure 4B). Furthermore, genes that significantly increased in expression following PCGF1 removal were more strongly enriched for polycomb target genes than those that significantly increased following KDM2 removal (Figure 4C). Genes showing increased expression following PCGF1 removal were a subset of those showing increased expression following KDM2 protein removal (Figure 4D), and there was only a moderate positive correlation between the gene expression changes in these lines (Supplementary Figure S4H). These observations indicate that a small proportion of the gene de-repression events in cells where KDM2 proteins are removed are related to the activity of the KDM2B–PRC1 complex.

Building on this important observation, we examined in more detail the gene expression changes that manifest from KDM2 protein removal from CGIs. From this it was evident that genes with low starting expression level more strongly increased in expression, including non-polycomb target genes (Figure 4E). Gene ontology analysis revealed that genes that significantly increased in expression were enriched for a variety of developmental terms (Figure 4F), consistent with some of the effects being related to the polycomb system, but also a variety of basic cellular processes that are unrelated (Figure 4G). This reflects the generalized increase in gene expression that occurs following KDM2 removal. Together, these observations reveal that KDM2 proteins play a widespread role in gene repression from CGI-associated gene promoters and do so largely through mechanisms that are independent of the polycomb repressive system.

### KDM2B plays the predominant role in gene repression

**Figure 5.**
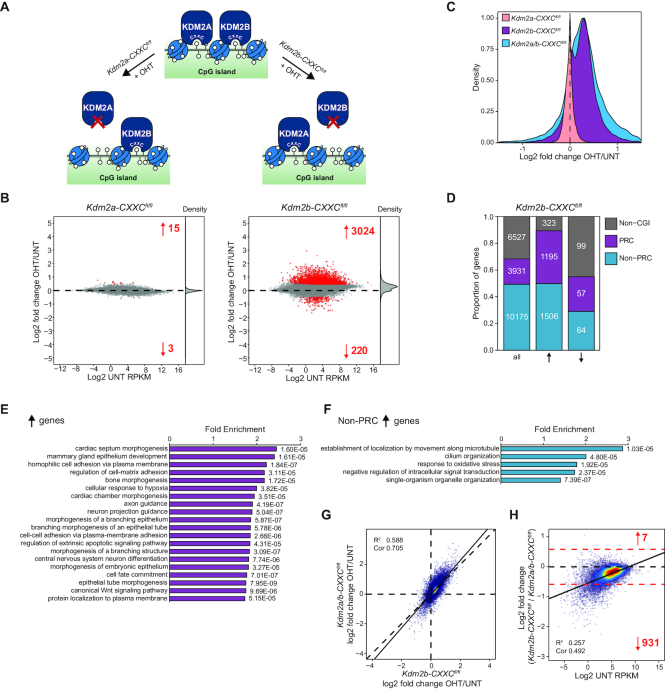
KDM2B plays the predominant role in gene repression. (**A**) A schematic of the *Kdm2a-CXXC^fl/fl^* and *Kdm2b-CXXC^fl/fl^* systems in which addition of tamoxifen (OHT) leads to the generation of KDM2A or KDM2B proteins that lack the ZF-CxxC domain, respectively, and therefore are unable to bind to chromatin. (**B**) MA-plots showing log2 fold change in gene expression (cnRNA-seq) in *Kdm2a-CXXC^fl/fl^* (left) or *Kdm2b-CXXC^fl/fl^* mESCs (right) following tamoxifen treatment. The number of genes with significantly increased or decreased expression (p-adj < 0.05 and > 1.4-fold) are shown in red and density of gene expression changes is shown on the right. (**C**) A density plot showing the distribution of the log2 fold change in gene expression following tamoxifen treatment of *Kdm2a-CXXC^fl/fl^, Kdm2b-CXXC^fl/fl^* or *Kdm2a/b-CXXC^fl/fl^* mESCs, for all genes. (**D**) A bar graph illustrating the distribution of genes between three gene classes (Non-CGI, Non-PRC, PRC) described in Figure 4C, for all genes and for genes that significantly increased or decreased in expression following tamoxifen treatment of *Kdm2b-CXXC^fl/fl^* mESCs. (**E**) Gene ontology analysis of genes that significantly increased in expression following tamoxifen treatment of *Kdm2b-CXXC^fl/fl^* mESCs. (**F**) As (**E**), but for the subset of significantly increasing genes that were not classified as polycomb target genes. (**G**) A scatter plot comparing the log2 fold change in gene expression (cnRNA-seq) following tamoxifen treatment of *Kdm2b-CXXC^fl/fl^* and *Kdm2a/b-CXXC ^fl/fl^* mESCs. The solid line shows the linear regression, and the coefficient of determination (*R*^2^) and Spearman correlation coefficient (Cor) are annotated. (**H**) A scatter plot of the log2 fold change in gene expression (cnRNA-seq) following tamoxifen treatment of *Kdm2a/b-CXXC ^fl/fl^* mESCs for genes that significantly increased in expression, plotted against the ratio of the log2 fold change in gene expression following tamoxifen treatment of *Kdm2b-CXXC^fl/fl^* versus *Kdm2a/b-CXXC^fl/fl^* mESCs. A 1.5-fold threshold (red dotted lines) was used to define genes which were differentially regulated between the two datasets, and the number of genes with more than 1.5-fold increased or decreased expression is shown in red. The solid line shows the linear regression, and the coefficient of determination (R^2^) and Spearman correlation coefficient (Cor) are annotated.

Loss of both KDM2A and KDM2B from CGI chromatin simultaneously resulted in widespread increases in gene expression (Figure 3). However, it was unclear from these experiments whether KDM2A, KDM2B or both contribute to gene repression. To examine this question, we developed a conditional mESC system in which we could remove KDM2A alone by tamoxifen-induced deletion of its ZF-CxxC domain (*Kdm2a-CXXC^fl/fl^*, Figure 5A, Supplementary Figures S5A and S3A). cnRNA-seq revealed that removal of KDM2A led to virtually no changes in gene expression (Figure 5B), indicating that KDM2A alone is not required to maintain normal gene expression in mESCs. We next investigated the contribution of KDM2B to the regulation of gene expression, performing cnRNA-seq using a *Kdm2b-CXXC^fl/fl^* mESC line (39) (Figure 5A and Supplementary Figure S5B). cnRNA-seq revealed that removal of KDM2B alone was sufficient to cause increases in the expression of thousands of genes (Figure 5B) and, unlike KDM2A removal, largely recapitulated the widespread increases in gene expression that occurred following KDM2A/B removal (Figure 5C). Genes that significantly increased in expression following KDM2B removal were enriched for CGI-associated genes (Figure 5D). Furthermore, gene ontology analysis revealed that these significantly increasing genes were enriched for a variety of developmental terms characteristic of polycomb target genes (Figure 5E) but also terms relating to basic cellular processes (Figure 5F). These observations suggest that removal of KDM2B alone, like removal of KDM2A/B together, leads to widespread increases in the expression of CGI-associated genes.

A comparison of the gene expression changes following KDM2B removal alone and KDM2A/B removal together revealed good overall correlation (Figure 5G), indicating that the gene expression changes following KDM2B removal largely recapitulated those following removal of KDM2A/B together. However, a more detailed analysis revealed 931 significantly increasing genes that less strongly increased in expression following loss of KDM2B compared to KDM2A/B together, and this set was enriched for genes with low expression level (Figure 5H). This suggests that KDM2A plays a role in restricting the expression of these genes following KDM2B removal. Together our findings demonstrate that KDM2B plays the predominant role in repressing gene expression, whilst KDM2A may cooperate with KDM2B to counteract expression at a subset of genes.

### KDM2 proteins regulate polymerase occupancy but not chromatin accessibility at CGIs

**Figure 6.**
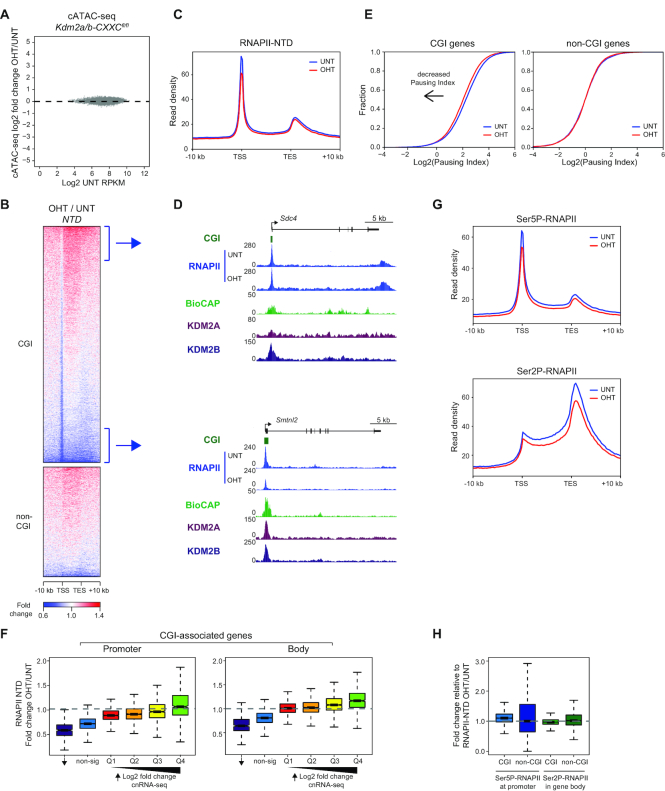
KDM2 proteins regulate RNAPII occupancy but not chromatin accessibility at CGI-associated gene promoters. (**A**) An MA-plot showing log2 fold change in the accessibility (cATAC-seq) of CGI-associated gene promoters in K*dm2a/b-CXXC^fl/fl^* mESCs following tamoxifen treatment. No promoters significantly changed in accessibility (p-adj < 0.05 and > 1.4-fold). (**B**) A heatmap of the fold change in RNAPII ChIP-seq signal following tamoxifen treatment of K*dm2a/b-CXXC^fl/fl^* mESCs, for CGI-associated (*n* = 14106) and non-CGI-associated (*n* = 6527) gene promoters. (**C**) A metaplot showing RNAPII enrichment at CGI-associated genes before (UNT) and after tamoxifen treatment (OHT) of *Kdm2a/b-CXXC^fl/fl^* mESCs. (**D**) Genomic snapshots showing RNAPII occupancy before (UNT) and after tamoxifen treatment (OHT) of *Kdm2a/b-CXXC^fl/fl^* mESCs, for (above) a representative gene that retains RNAPII at the promoter and increases in RNAPII occupancy throughout the gene body, and (below) a representative gene that decreases in RNAPII at both promoter and gene body regions. BioCAP and KDM2A and KDM2B ChIP-seq signal are shown for reference (63). (**E**) Empirical cumulative density function (ECDF) plots of RNAPII pausing index for CGI-associated (left) or non-CGI-associated (right) genes, before (UNT) and after tamoxifen treatment (OHT) of *Kdm2a/b-CXXC^fl/fl^* mESCs. (**F**) Boxplots showing the fold change in RNAPII occupancy at CGI-associated gene promoters (left) or gene bodies (right) following tamoxifen treatment of *Kdm2a/b-CXXC^fl/fl^* mESCs, for CGI-associated genes that significantly decreased in expression, with no significant change in expression (‘non-sig’), or that significantly increased in expression. Genes that significantly increased in expression were separated into quartiles according to their log2 fold change in expression (Q1 < Q2 < Q3 < Q4). (**G**) Metaplots showing Ser5P-RNAPII (upper panel) or Ser2P-RNAPII (lower panel) enrichment at CGI-associated genes before (UNT) and after tamoxifen treatment (OHT) of *Kdm2a/b-CXXC^fl/fl^* mESCs. (**H**) A boxplot showing the fold change in Ser5P-RNAPII at gene promoters (left) or Ser2P-RNAPII at gene bodies (right) following tamoxifen treatment of *Kdm2a/b-CXXC^fl/fl^* mESCs, normalized to RNAPII-NTD signal. The fold changes for CGI-associated and non-CGI-associated genes are shown.

Gene regulatory elements and gene promoters are characterized by elevated chromatin accessibility (70–72), and this is thought to play an important role in regulating gene expression. Accessibility at CGI-associated gene promoters broadly correlates with transcriptional output, with the promoters of highly transcribed CGI-associated genes being more highly accessible (73). Therefore we wondered whether the increases in gene expression following removal of KDM2 proteins resulted from increases in the accessibility at CGI-associated gene promoters in the absence of KDM2A/B. To test this we carried out cATAC-seq following removal of KDM2 proteins. Importantly, we did not observe any significant change in the accessibility of CGI-associated gene promoters (Figure 6A; Supplementary Figure S6A and B), indicating that expression changes must manifest through effects on transcription that are independent of chromatin accessibility.

To examine this possibility in more detail, we carried out cChIP-seq for RNAPII before and after removal of KDM2 proteins. This revealed on average a widespread decrease in RNAPII occupancy at the TSSs of CGI-associated genes (Figure 6B and C), which was reproducible between replicates (Supplementary Figure S6C). In the gene body, alterations in RNAPII occupancy appeared to be related to the level of RNAPII reduction at the gene promoter. Genes that retained promoter-associated RNAPII showed increased RNAPII in the gene body and promoters showing reduced RNAPII levels also had moderately reduced RNAPII in the gene body (Figure 6D). Importantly, these effects were restricted to CGI-associated genes, in agreement with the function of KDM2A/B at CGIs. To examine in more detail the nature of the defects in RNAPII function at CGI-associated gene promoters, we calculated the RNAPII pausing index, which is often used a proxy for RNAPII pause release (Supplementary Figure S6D). This showed a modest but clear decrease in pausing index when KDM2 proteins were removed (Figure 6E and Supplementary Figure S6E), which we also validated by ChIP-qPCR (Supplementary Figure S6F) and importantly this effect was not observed for non-CGI-associated genes. This suggests that removal of KDM2 proteins may contribute to an increased rate of RNAPII pause release from CGI-associated gene promoters. A comparison of the changes in RNAPII occupancy with the gene expression changes following KDM2 removal revealed a moderate positive correlation (Supplementary Figure S6G). Genes that decreased in expression lost RNAPII from both their promoter and gene body, consistent with their reduced transcriptional activity (Figure 6F and Supplementary Figure S6H). In contrast, the genes that most strongly increased in expression retained RNAPII at their promoter and had elevated RNAPII occupancy throughout the gene body. These effects on RNAPII could be explained by an increase in the rate of transcription initiation at genes that show large increases in expression which, when combined with an increased rate of pause release, results in the accumulation of RNAPII throughout the gene body and increases in transcript levels. In contrast, there was a dramatic reduction of RNAPII occupancy at both the promoter and body of genes which decreased in expression, consistent with their reduced transcription. Importantly, the change in the distribution of Ser5-phosphorylated RNAPII, which is enriched at promoter regions, and Ser2-phosphorylated RNAPII, which is enriched throughout gene bodies, resembled that of total RNAPII (Supplementary Figure S6I). There was no obvious shift in the position of either Ser2- or Ser5-phosphorylated RNAPII enrichment throughout CGI-associated genes (Figure 6G), and only minor changes in the relative enrichment of Ser5- or Ser2-phosphorylated RNAPII compared to total RNAPII at CGI gene promoters and gene bodies, respectively (Figure 6H). Together our findings suggest that KDM2 proteins play a role in regulating RNAPII activity at gene promoters, potentially by limiting initiation and pause release to constrain productive transcription from these regions of the genome.

## DISCUSSION

Chromatin modifying complexes are thought to play central roles in regulating gene expression through their enzymatic activities. Yet, for most of these complexes, the importance of their histone modifying activities in gene regulation remains to be tested. KDM2 histone demethylases have been proposed to contribute to an H3K36me2-depleted and transcriptionally permissive chromatin state at CGI-associated gene promoters (8,9). Alternatively, KDM2 demethylase activity has also been suggested to contribute to gene repression in some specific instances (19,33–37). However, the extent to which KDM2 proteins regulate gene expression and how this is related to their H3K36me2 demethylase activity has remained untested. Here, using combinatorial genetic perturbation and detailed genome-wide approaches, we discover that the histone demethylase activity of KDM2 proteins contributes modestly to the H3K36me2 depletion at CGI-associated gene promoters (Figure 1) and has minimal effects on gene expression (Figure 2). In contrast, using calibrated gene expression analysis we discover an unexpectedly widespread histone demethylase-independent role for KDM2 proteins in constraining the expression of CGI-associated genes (Figure 3). Importantly, repression by KDM2 proteins is not limited to polycomb target genes, which are known to be regulated by the KDM2B–PRC1 complex (Figure 4). Nevertheless, we find that KDM2B plays the predominant role in repressing expression, whilst KDM2A appears to contribute at a subset of genes (Figure 5). Finally, the effects of KDM2 proteins on gene expression are not mediated through changes in chromatin accessibility, but instead KDM2 proteins appear to play a role in constraining RNAPII occupancy and possibly pause release at CGI-associated gene promoters to limit transcription (Figure 6). Together, this demonstrates that KDM2 proteins regulate gene expression independently of their histone demethylase activity and through mechanisms that appear to regulate RNAPII function at CGI-associated gene promoters. These discoveries reveal an interesting new chromatin modification-independent role for CGIs and the KDM2 proteins in constraining gene expression.

Our understanding of how histone modification states are specified and regulated remains poorly understood. In the context of histone H3K36, NSD1–3 and ASH1L, the main H3K36me1/2 methyltransferases, can associate with gene promoters and genic regions, and H3K36me2 blankets most of the genome (74–79). However, our genome-wide profiling of H3K36me2 reveals that the bodies of highly transcribed genes and CGI-associated gene promoters are exceptions to this, being uniquely depleted of H3K36me2. This suggests that mechanisms must function to shape H3K36me2 at distinct regions of the genome. The depletion of H3K36me2 in highly transcribed gene bodies is likely due to conversion to H3K36me3 by the SETD2 protein, which interacts with RNAPII and functions as an H3K36 trimethyltransferase in gene bodies during transcriptional elongation (80–88). We and others had previously proposed that the H3K36me2-depleted state at CGI-associated gene promoters relies on KDM2 proteins to actively remove H3K36me2 from these regions. Now, using a cell system where we can induce the removal of KDM2 demethylase activity, we show that KDM2 enzymes contribute modestly to depletion of H3K36me2 at CGI-associated gene promoters. This suggests that the activity of NSD/ASH1L may be inhibited, or that other histone demethylases may compensate for the loss of KDM2 enzymes, at these regions of the genome. The latter of these two possibilities seems the most likely, as KDM4A-C demethylases catalyse the removal of H3K36me2/3 (89–92) and also associate broadly with gene promoters (93,94). It is also possible that KDM2 demethylases initiate H3K36me2-depletion at CGIs and other mechanisms, possibly including KDM4 demethylases, are required for its maintenance. Therefore, determining whether the H3K36me2-depleted state at CGI-associated gene promoters results from active removal of this modification and contributes to gene regulation awaits further investigation, including combinatorial removal of KDM2 and KDM4 demethylase activity.

When studied in the context of individual genes, KDM2A and KDM2B have been proposed to function in both gene activation and repression. However, which of these activities is most prevalent and whether these paralogous proteins function together to achieve appropriate gene regulation have remained unknown. Using combinatorial inducible genetic perturbation strategies and calibrated RNA-seq we now reveal that KDM2 proteins function primarily as repressors of gene expression and elicit their effects via a demethylase-independent mechanism. Gene repression by KDM2 proteins is remarkably widespread but largely restricted to CGI-associated genes, in agreement with the occupancy of KDM2 proteins at these regions of the genome through their ZF-CxxC DNA binding domain. However, lowly expressed genes were more susceptible to increases in gene expression when KDM2 proteins were removed. Therefore, KDM2 proteins may function to generically constrain transcription from CGI-associated gene promoters, but only function to counteract low-level activation signals. In agreement with this suggestion, CGI-associated genes that are already highly expressed are largely unaffected by KDM2 loss, despite the fact that KDM2 proteins occupy their promoters. In the context of these observations, we propose that the repressive activity of KDM2 proteins may effectively create a CGI-imposed barrier to gene activation which protects against low-level or inappropriate gene activation signals. This could be particularly important in the context of cellular differentiation where excessive gene expression noise or precocious gene activation may have deleterious consequences for the highly orchestrated cascade of gene expression events that lead to appropriate acquisition of new cell fates. Indeed, whilst embryoid body formation was largely unperturbed following loss of KDM2 demethylase activity (Supplementary Figure S7A), removal of KDM2 proteins from chromatin led to major gene expression defects and the failure to appropriately induce the expression of a variety of developmental genes (Supplementary Figure S7B). This may be the consequence of inappropriate gene expression that manifests in the ESC state that precludes induction of a normal differentiation programme.

Gene repression by the KDM2 proteins occurs independently of their JmjC domain and histone demethylase activity, raising the interesting question of how they repress gene expression. By examining the binding of RNAPII and its modified forms throughout the genome following loss of KDM2 proteins, we discover that there is a widespread reduction in RNAPII occupancy at CGI-associated gene promoters. This suggests that one activity of KDM2 proteins at CGIs may be to constrain productive transcription, perhaps through a process that directly regulates RNAPII pause release. It is intriguing to note that, on average, RNAPII occupancy moderately decreased in gene bodies following loss of KDM2 proteins, despite a widespread increase in gene expression. This raises the possibility that there are further alterations to RNAPII behaviour, such as elongation rate, following KDM2 removal. Based on these observations, an important area of future work will be to examine the mechanisms by which KDM2 proteins affect RNAPII activity and to determine how direct this is.

We speculate that one mechanism by which KDM2 proteins could potentially modulate RNAPII-dependent transcription processes is through ubiquitylation. This is because, in addition to their JmjC domains, KDM2 proteins also encode FBOX and LRR domains. FBOX domains bind a protein called SKP1, and we and others have previously shown that KDM2A and KDM2B both interact with SKP1 (15,40,95,96). SKP1 is a central component of SCF-type E3 ubiquitin ligase complexes (97), whilst FBOX-containing proteins are thought to confer substrate specificity for SCF complexes through additional domains such as the LRR domain (98). This suggests that KDM2 proteins might identify target proteins for ubiquitylation. Indeed, KDM2B was reported to ubiquitylate the transcription factor c-Fos, leading to its degradation by the proteasome (99). KDM2A has also been proposed to possess E3 ligase activity, as its overexpression stimulates 53BP1 ubiquitylation (100). The specificity of these putative KDM2 E3 ubiquitin ligase complexes remains to be investigated. However, given that KDM2 proteins act broadly to repress gene expression and may regulate RNAPII activity, one might envisage that KDM2 proteins could regulate a component of the core transcriptional machinery or another general modulator of gene transcription. Therefore, in future work it will be interesting to explore whether KDM2 proteins have a role in proteostasis at CGIs and to understand whether this contributes to their function in the repression of gene expression and the regulation of RNAPII activity.

In conclusion, we discover that KDM2 proteins are CGI-specific transcriptional repressors that appear to function to constrain low-level gene activation signals. Interestingly, DNA situated in CGIs is known to be highly accessible, differentiating it from much of the rest of the genome. It has been proposed that this accessibility highlights the location of gene regulatory elements within large and complex vertebrate genomes, and allows transcriptional regulators and the transcriptional machinery to more easily access the underlying DNA and enable gene expression. However, an unintended consequence of this CGI-associated accessibility may be that it renders these regions susceptible to low-level and potentially inappropriate gene activation signals. We speculate that, in response to this potentially deleterious side effect of CGI accessibility, KDM2 proteins may have evolved to bind CGIs and constrain transcription. Indeed, we show that loss of KDM2 proteins does not affect accessibly at CGIs but does broadly affect gene expression and RNAPII occupancy. Therefore we propose that CGIs create an appropriate balance of transcriptionally permissive and restrictive activities to help control gene expression.

## DATA AVAILABILITY

Datasets have been deposited in the Gene Expression Omnibus (GEO) with accession number GSE126864.

## Supplementary Material

gkz607_Supplemental_File
