# Supplementary material for: KDM2 proteins constrain transcription from CpG island gene promoters independently of their histone demethylase activity
Source: Nucleic Acids Res. 2019 Jul 31;47(17):9005–23. doi: 10.1093/nar/gkz607 (PMC6753492; doi:10.1093/nar/gkz607)
Supplement: gkz607_Supplemental_File [file gkz607_supplemental_file.pdf]

SUPPLEMENTARY FIGURES

Supplementary Figure 1 – Related to Figure 1.

(A) A schematic representation of the *Kdm2a/b-JmjC*$^{fl/fl}$ mESC line, in which loxP sites were inserted into the *Kdm2a* and *Kdm2b* genes flanking exons that encode the JmjC domain.

(B) ChIP-qPCR analysis showing KDM2A (upper panel) and KDM2B (lower panel) enrichment relative to input in *Kdm2a/b-JmjC*$^{fl/fl}$ mESCs before (UNT) and after tamoxifen treatment (OHT). Error bars show standard error of the mean of three biological replicates.

(C) Quantitation of western blots of histone extract from *Kdm2a/b-JmjC*$^{fl/fl}$ mESCs, before (UNT) and after 96 hours of tamoxifen treatment (OHT). Signal is normalised to histone H4 and is represented relative to average UNT signal. Error bars show standard deviation of three biological replicates. Significance was tested using a Student's T-test (non-significant (ns) if $p > 0.05$).

(D) Representative western blots. Histone H4 is shown as a loading control.

(E) Genomic snapshots of CGI-associated gene promoters showing normalised H3K36me2 ChIP-seq signal before (UNT) and after tamoxifen treatment (OHT) of *Kdm2a/b-JmjC*$^{fl/fl}$ mESCs.

(F) ChIP-qPCR analysis showing H3K36me2 enrichment relative to input in *Kdm2a/b-JmjC*$^{fl/fl}$ mESCs before (UNT) and after tamoxifen treatment (OHT). Error bars show standard error of the mean of three biological replicates.

(G) Boxplots showing CpG density (left) and size (right) of intragenic and promoter-associated CGIs.

(H) A metaplot of cnRNA-seq signal in K*dm2a/b-JmjC*$^{fl/fl}$ mESCs, for CGI-associated genes separated into quartiles according to their expression level (Q1 < Q2 < Q3 < Q4) and for non-CGI-associated genes. Genes were scaled to the same length and aligned at their TSS and TES.

(I) A metaplot showing normalised H3K36me2 ChIP-seq signal for highly expressed CGI-associated genes (Q4, as above), excluding genes that are less than 3 kb in length.

(J) A scatter plot comparing the enrichment of H3K36me2 and H3K36me3 in the gene body of highly expressed CGI-associated genes (Q4, as above) (1). The solid line shows the linear regression, and the coefficient of determination (R^2) and Spearman correlation coefficient (Cor) are annotated.

Supplementary Figure 2 – Related to Figure 2.

(A) A metaplot showing enrichment of ATAC-seq signal at the TSS of all CGI-associated genes (n=14106) before (UNT, blue) and after tamoxifen treatment (OHT, red) of *Kdm2a/b-JmjC*$^{fl/fl}$ mESCs.

Supplementary Figure 3 – Related to Figure 3.

(A) A schematic representation of the *Kdm2a/b-CXXC*$^{fl/fl}$ mESC line, in which loxP sites were inserted into the *Kdm2a* and *Kdm2b* genes flanking exons that encode the ZF-CxxC domain. sgRNA.1 and sgRNA.2 indicate the position of CRISPR-mediated loxP insertion.

(B) ChIP-qPCR analysis showing KDM2A (left panel) and KDM2B (right panel) enrichment relative to input in *Kdm2a/b-CXXC$^{fl/fl}$* mESCs before (UNT) and after tamoxifen treatment (OHT). Error bars show standard error of the mean of three biological replicates.

(C) ChIP-qPCR analysis showing H3K36me2 enrichment relative to input in *Kdm2a/b-CXXC$^{fl/fl}$* mESCs before (UNT) and after tamoxifen treatment (OHT). Error bars show standard error of the mean of three biological replicates.

(D) Droplet digital PCR analysis, showing the fold change in template cDNA concentration following tamoxifen treatment (OHT) of *Kdm2a/b-CXXC$^{fl/fl}$* mESCs (blue). Error bars show standard deviation of three biological replicates. The fold change calculated by cnRNA-seq is shown for comparison (green), and the significance of this change is annotated below the graph. The dashed lines represent no change in expression (black) and the 1.4 fold change threshold used for cnRNA-seq analysis (red).

(E) An MA-plot showing log2 fold change in gene expression in *Kdm2a/b-CXXC$^{fl/fl}$* mESCs following tamoxifen treatment (OHT), normalising nRNA-seq data to total library size. The number of genes with significantly increased or decreased expression (p-adj < 0.05 and > 1.4-fold) is shown in red and density of gene expression changes is shown on the right.

(F) A bar graph comparing the distribution of genes into three classes – non-CGI, polycomb (PRC) occupied and non-PRC occupied – for all genes and for genes that significantly decreased in expression following tamoxifen treatment of *Kdm2a/b-CXXC$^{fl/fl}$* mESCs. Non-CGI genes are genes that lack a CGI at their promoter. Non-PRC-occupied genes have a CGI promoter that is not bound by polycomb complexes, while PRC-occupied genes have a CGI promoter that is bound by polycomb complexes.

Supplementary Figure 4 – Related to Figure 4.

(A) Metaplots showing enrichment of H2AK119ub1 and H3K27me3 ChIP-seq signal at the TSS of all CGI-associated genes (n=14106, green) and of the subset of these genes that significantly increased in expression following tamoxifen treatment of *Kdm2a/b-CXXC$^{fl/fl}$* mESCs (n=3879, red).

(B) Genomic snapshots of typical polycomb target genes showing cnRNA-seq and ChIP-seq for H2AK119ub1, H3K27me3, KDM2A and KDM2B before (UNT) and after tamoxifen treatment (OHT) of *Kdm2a/b-CXXC$^{fl/fl}$* mESCs.

(C) Metaplots showing enrichment of H2AK119ub1 and H3K27me3 ChIP-seq signal at the TSS of all CGI-associated genes (n=14106) before (UNT, blue) and after tamoxifen treatment of *Kdm2a/b-CXXC$^{fl/fl}$* mESCs (OHT, red).

(D) ChIP-qPCR analysis showing H3K27me3 enrichment relative to input in *Kdm2a/b-CXXC$^{fl/fl}$* mESCs before (UNT) and after tamoxifen treatment (OHT). Error bars show standard error of the mean of three biological replicates.

(E) Metaplots showing enrichment of H2AK119ub1 and H3K27me3 ChIP-seq signal at the TSS of all CGI-associated genes (n=14106) before (UNT, blue) and after tamoxifen treatment of *Kdm2a/b-JmjC$^{fl/fl}$* mESCs (OHT, red).

(F) Genomic snapshots of typical polycomb target genes showing cnRNA-seq and ChIP-seq for H2AK119ub1 and H3K27me3 before (UNT) and after tamoxifen treatment (OHT) of *Kdm2a/b-JmjC$^{fl/fl}$* mESCs. ChIP-seq for KDM2A and KDM2B is shown for reference.

(G) ChIP-qPCR analysis showing H3K27me3 enrichment relative to input in *Kdm2a/b-JmjC$^{fl/fl}$* mESCs before (UNT) and after tamoxifen treatment (OHT). Error bars show standard error of the mean of three biological replicates.

(H) A scatter plot comparing the log2 fold change in gene expression (cnRNA-seq) following tamoxifen treatment of *Kdm2a/b-CXXC$^{fl/fl}$* and *Pcgf1$^{fl/fl}$* mESCs. The solid line shows the linear regression, and the coefficient of determination (R^2) and Spearman correlation coefficient (Cor) are annotated.

Supplementary Figure 5 – Related to Figure 5.

(A) Western blot analysis for KDM2A and KDM2B in K*dm2a-CXXC$^{fl/fl}$* mESCs before (UNT) and after 96 hours of tamoxifen treatment (OHT). BRG1 is shown as a loading control for both blots. Asterisks indicate non-specific bands.

(B) As (A) but for K*dm2b-CXXC$^{fl/fl}$* mESCs.

Supplementary Figure 6 – Related to Figure 6.

(A) MA-plots showing log2 fold change in the accessibility (cATAC-seq) of CGI-associated gene promoters in K*dm2a-CXXC$^{fl/fl}$* (left) or K*dm2a-CXXC$^{fl/fl}$* (right) mESCs following tamoxifen treatment. No promoters significantly changed in accessibility (p-adj < 0.05 and > 1.4-fold).

(B) Metaplots showing enrichment of cATAC-seq signal at the TSS of all CGI-associated genes (n=14106) before (UNT, blue) and after tamoxifen treatment (OHT, red) of K*dm2a/b-CXXC$^{fl/fl}$* (left), K*dm2a-CXXC$^{fl/fl}$* (middle), and K*dm2b-CXXC$^{fl/fl}$* (right) mESCs.

(C) Metaplots showing RNAPII enrichment at CGI-associated genes before (UNT) and after tamoxifen treatment (OHT) of *Kdm2a/b-CXXC$^{fl/fl}$* mESCs, for three individual biological replicates.

(D) An illustration of the pausing index, the ratio of the average read density of RNAPII-NTD at the promoter and the average read density of RNAPII in the gene body.

(E) Scatter plots comparing the fold change in pausing index with the fold change in RNAPII occupancy at CGI-associated gene promoters (left) or gene bodies (right), following tamoxifen treatment of *Kdm2a/b-CXXC$^{fl/fl}$* mESCs. The solid line shows the linear regression, and the coefficient of determination (R^2) and Spearman correlation coefficient (Cor) are annotated.

(F) A bar graph showing the fold change in pausing index following tamoxifen treatment of *Kdm2a/b-CXXC$^{fl/fl}$* mESCs measured by RNAPII ChIP-qPCR at gene promoter and body regions (blue) or by RNAPII ChIP-seq (red). Error bars show standard error of the mean of three biological replicates.

(G) Scatter plots comparing the log2 fold change in gene expression (cnRNA-seq) with the log2 fold change in RNAPII occupancy (ChIP-seq) following tamoxifen treatment of *Kdm2a/b-CXXC$^{fl/fl}$* mESCs, at CGI-associated gene promoters (left) or gene bodies (right). The solid line

shows the linear regression, and the coefficient of determination (R^2) and Spearman correlation coefficient (Cor) are annotated.

(H) Metaplots showing RNAPII enrichment before (UNT) and after tamoxifen treatment (OHT) of *Kdm2a/b-CXXC*$^{fl/fl}$ mESCs, for significantly downregulated CGI-associated genes (left) and for the top quartile of significantly upregulated CGI-associated genes (right).

(I) Heatmap analyses of the fold change in RNAPII, Ser5-RNAPII or Ser2P-RNAPII ChIP-seq signal following tamoxifen treatment of K*dm2a/b-CXXC*$^{fl/fl}$ mESCs, for CGI-associated (n=14106) and non-CGI-associated (n=6527) gene promoters.

Supplementary Figure 7

(A) RT-qPCR gene expression analysis of developmental genes during an embryoid body differentiation time course, using *Kdm2a/b-JmjC*$^{fl/fl}$ mESCs before (UNT) or after tamoxifen treatment (OHT). Expression is shown relative to an average of two house-keeping genes. Error bars show standard error of the mean of three biological replicates.

(B) As (A), but for *Kdm2a/b-CXXC*$^{fl/fl}$ mESCs.

REFERENCES

1. Brookes, E., de Santiago, I., Hebenstreit, D., Morris, K.J., Carroll, T., Xie, S.Q., Stock, J.K., Heidemann, M., Eick, D., Nozaki, N. *et al.* (2012) Polycomb associates genome-wide with a specific RNA polymerase II variant, and regulates metabolic genes in ESCs. *Cell stem cell*, **10**, 157-170.
2. Long, H.K., Sims, D., Heger, A., Blackledge, N.P., Kutter, C., Wright, M.L., Grutzner, F., Odom, D.T., Patient, R., Ponting, C.P. *et al.* (2013) Epigenetic conservation at gene regulatory elements revealed by non-methylated DNA profiling in seven vertebrates. *eLife*, **2**, e00348.
3. Fursova, N.A., Blackledge, N.P., Nakayama, M., Ito, S., Koseki, Y., Farcas, A.M., King, H.W., Koseki, H. and Klose, R.J. (2019) Synergy between Variant PRC1 Complexes Defines Polycomb-Mediated Gene Repression. *Molecular cell*, **74**, 1020-1036.e1028.

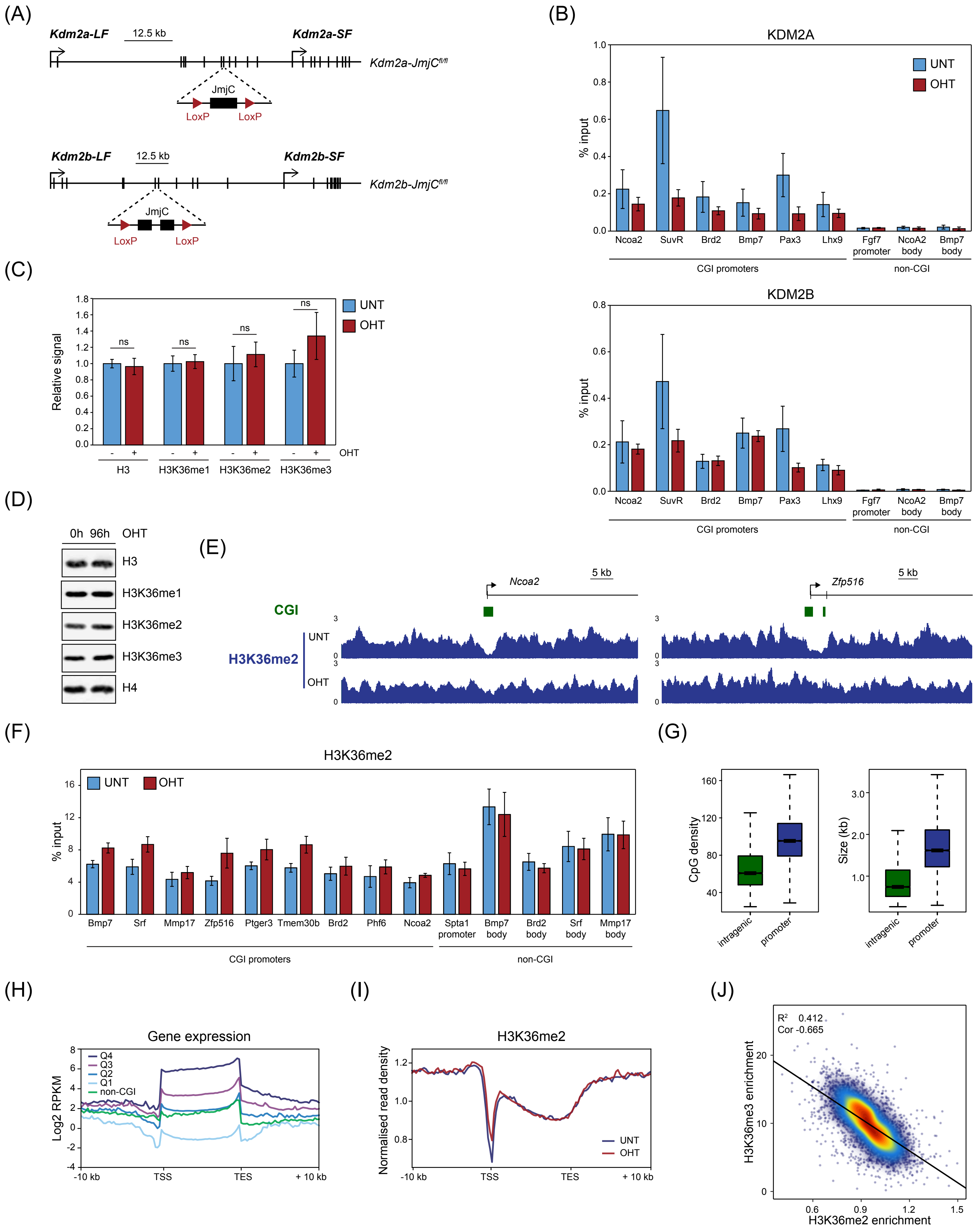

Supplementary Figure 1

(A)

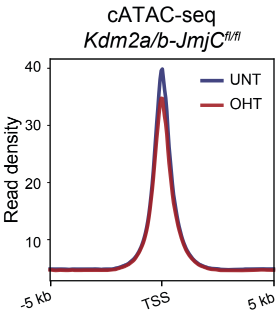

Supplementary Figure 2

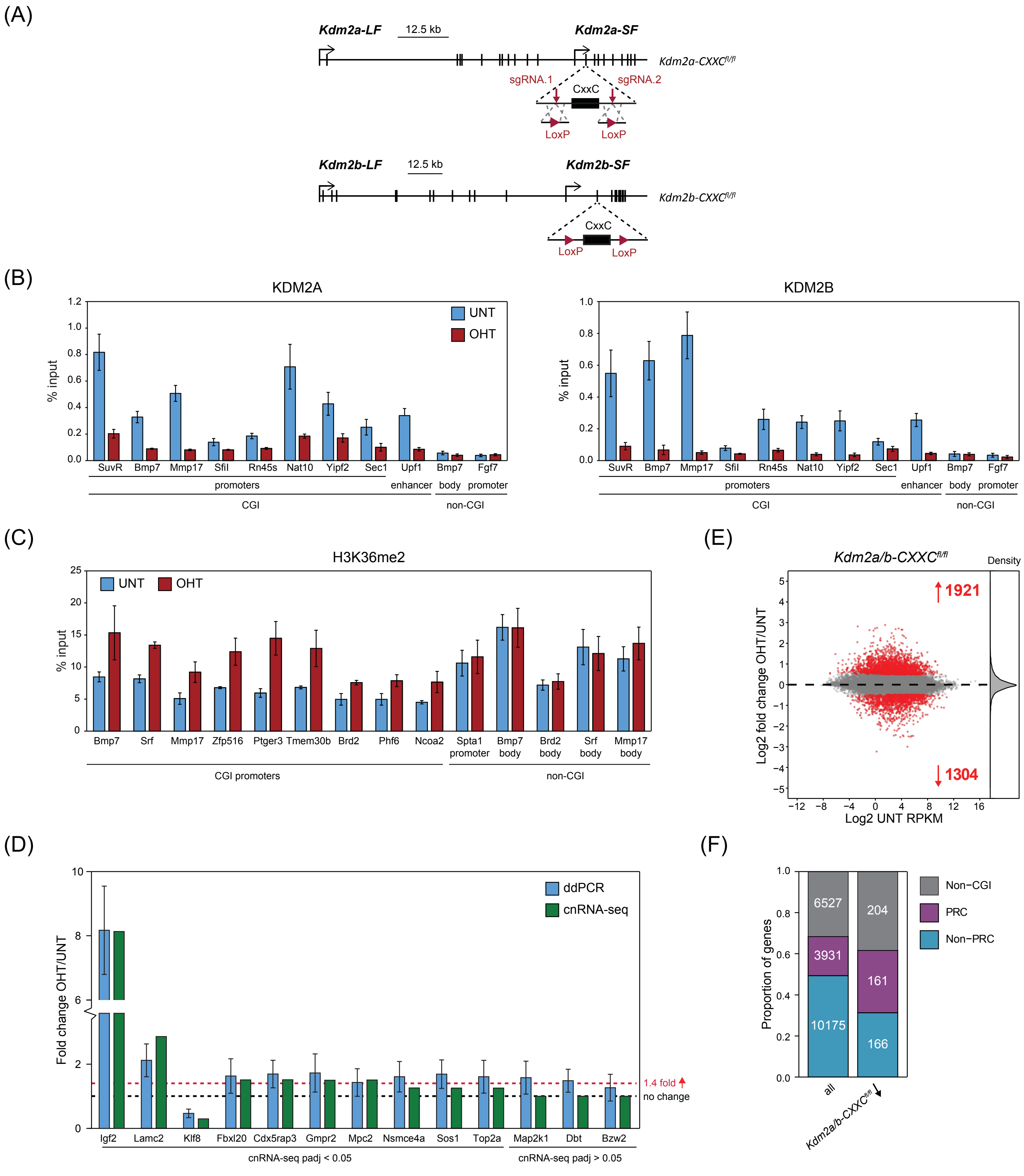

Supplementary Figure 3

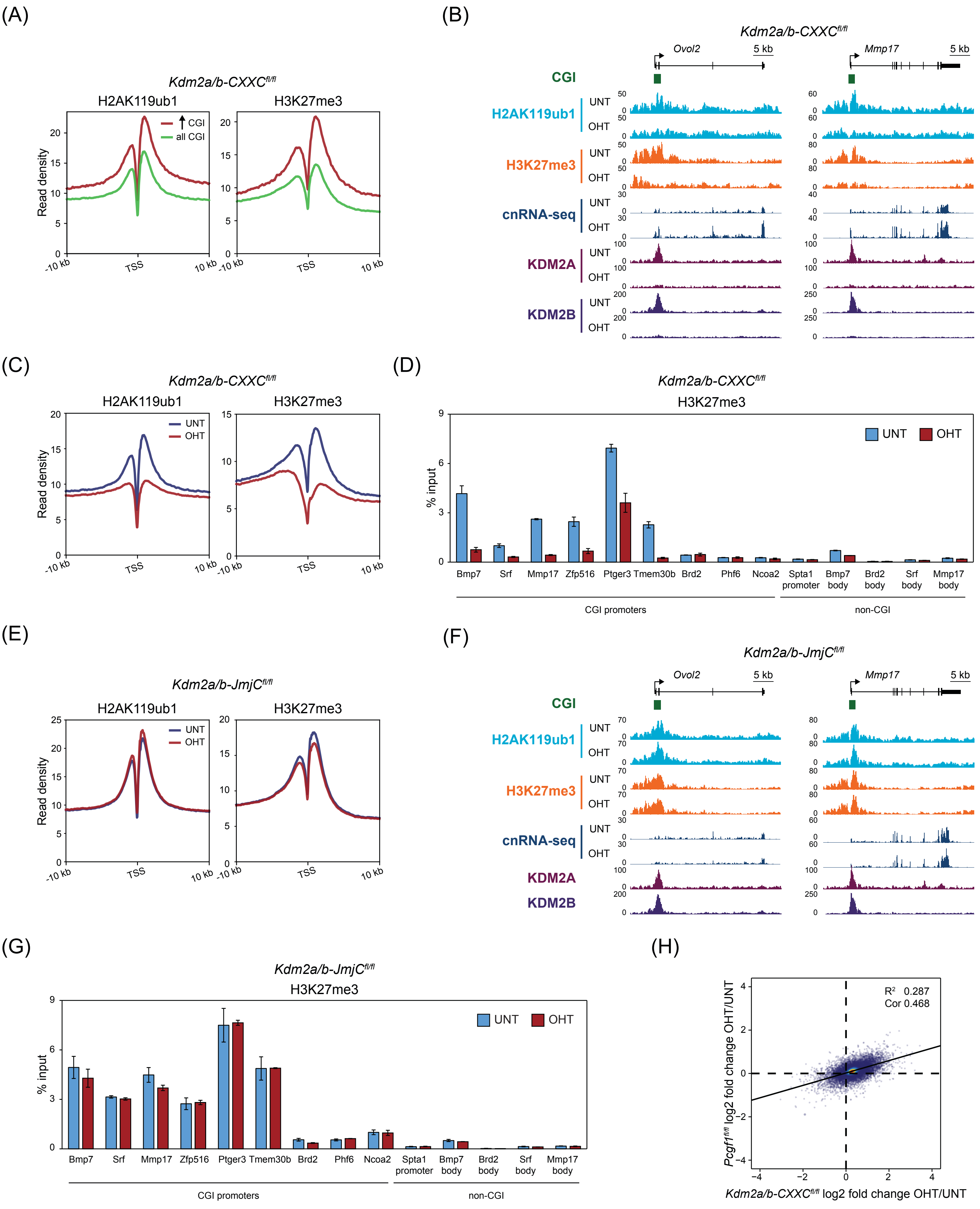

Supplementary Figure 4

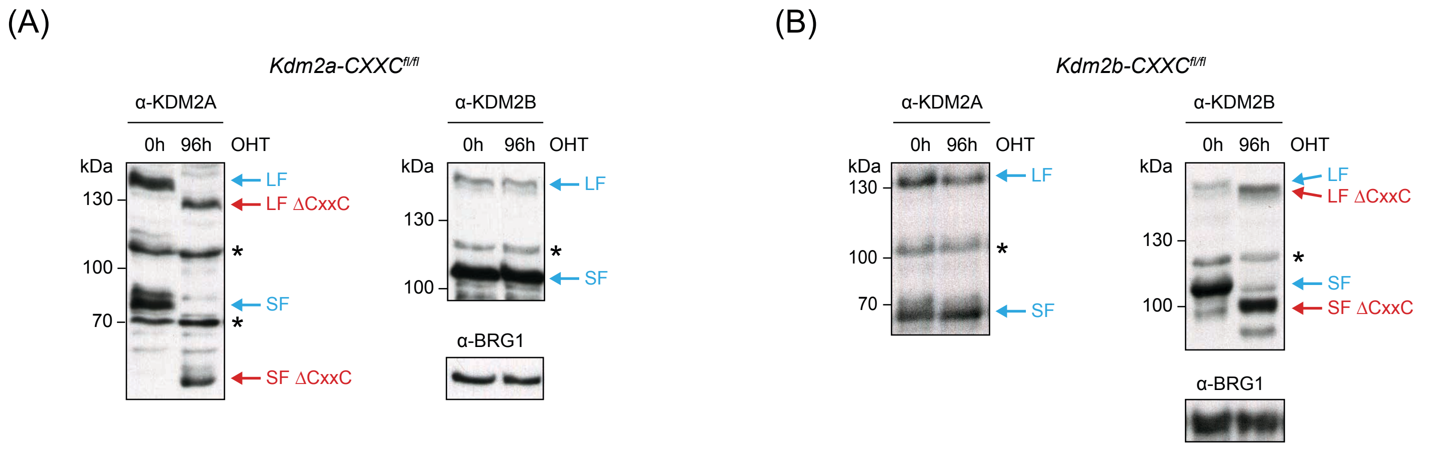

Supplementary Figure 5

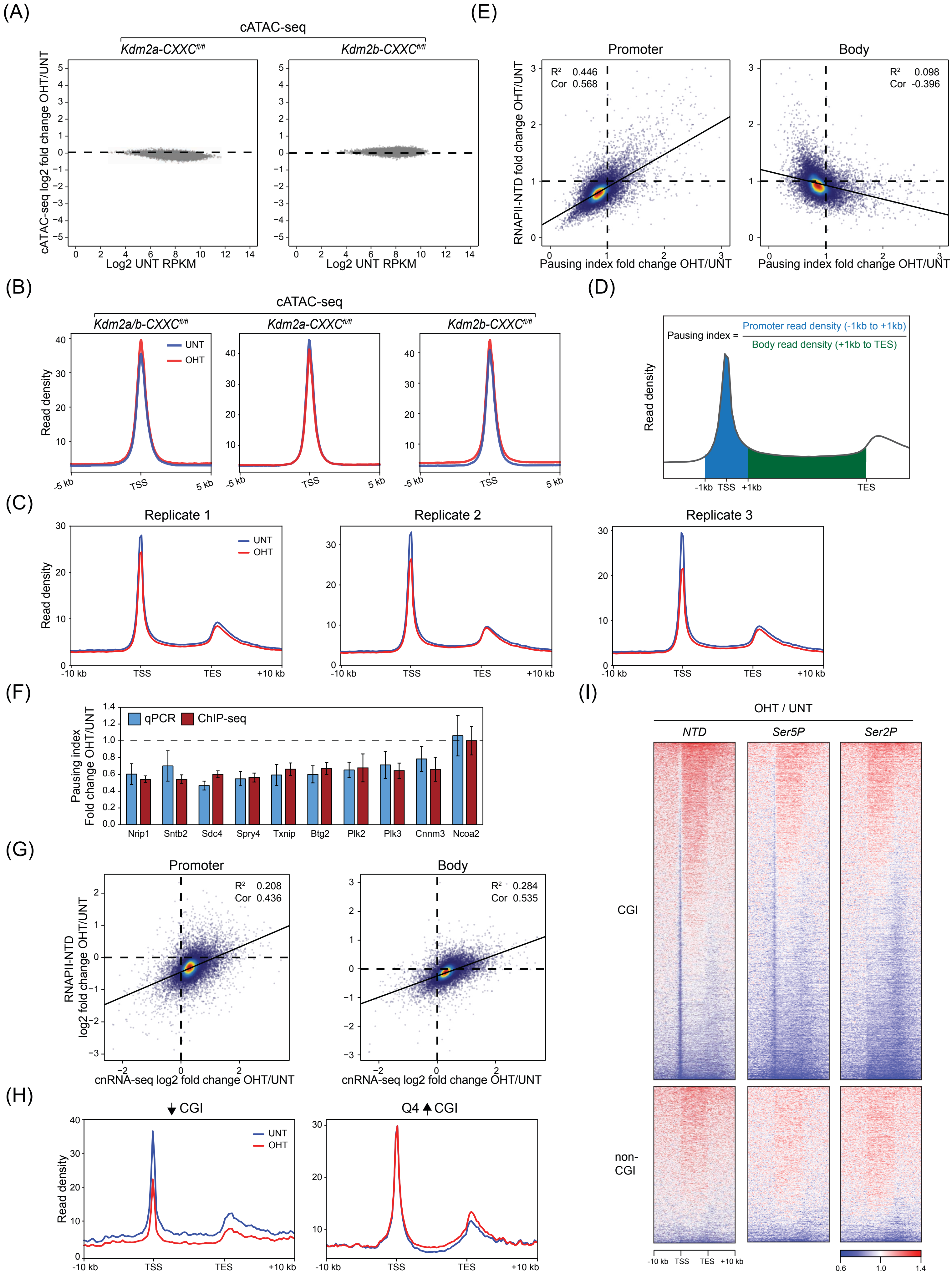

Supplementary Figure 6

(A)

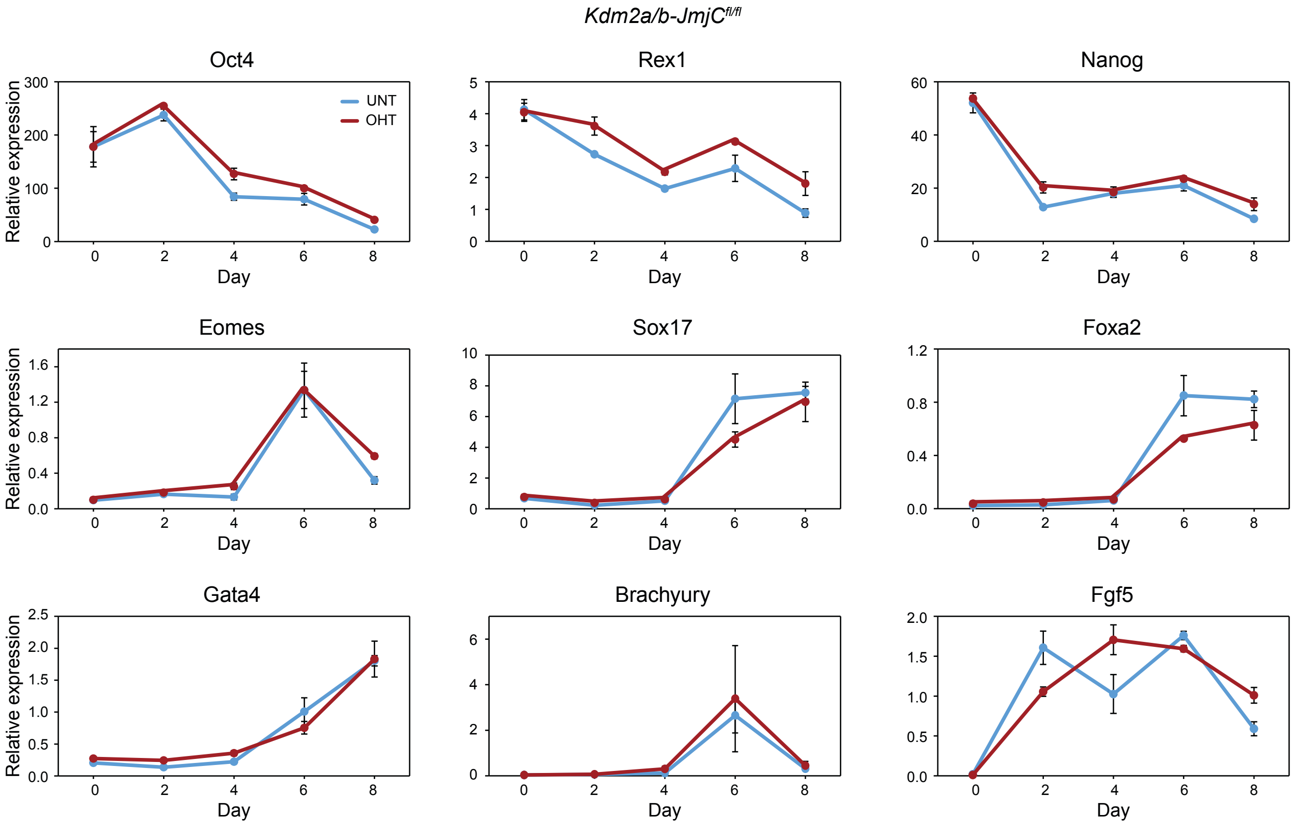

(B)

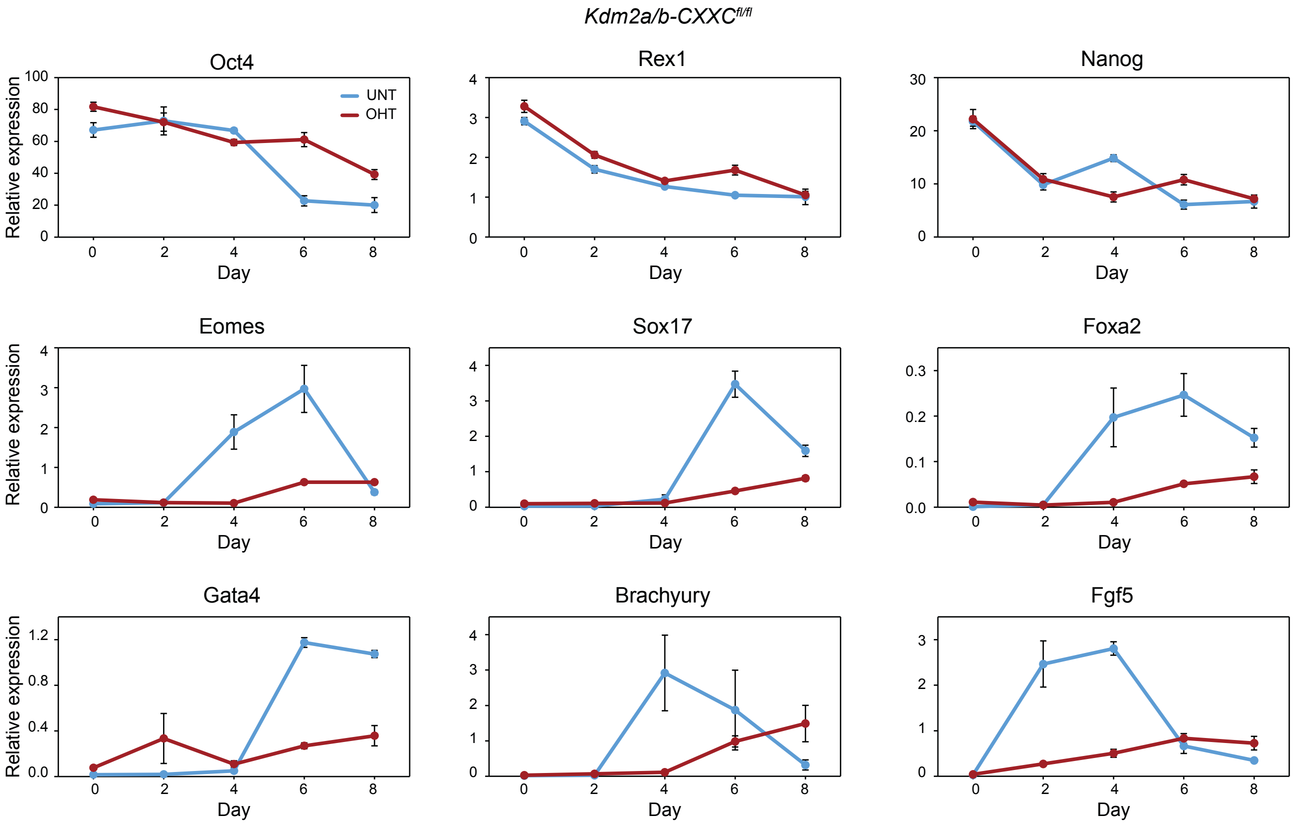

Supplementary Figure 7
